# A generalized supervised contrastive learning framework for integrative multi-omics prediction models

**DOI:** 10.3389/frmbi.2026.1825540

**Published:** 2026-08-13

**Authors:** Sen Yang, Shidan Wang, Yiqing Wang, Ruichen Rong, Bo Li, Andrew Y. Koh, Guanghua Xiao, Qiwei Li, Dajiang Liu, Xiaowei Zhan

**Affiliations:** 1 Department of Public Health Sciences, College of Medicine, Pennsylvania State University, Hershey, PA, United States; 2 Quantitative Biomedical Research Center, Department of Population and Data Sciences, University of Texas Southwestern Medical Center, Dallas, TX, United States; 3 Department of Statistics and Data Science, Southern Methodist University, Dallas, TX, United States; 4 Department of Pathology and Laboratory Medicine, University of Pennsylvania, Philadelphia, PA, United States; 5 Harold C. Simmons Cancer Center, University of Texas Southwestern Medical Center, Dallas, TX, United States; 6 Department of Microbiology, University of Texas Southwestern Medical Center, Dallas, TX, United States; 7 Department of Pediatrics, University of Texas Southwestern Medical Center, Dallas, TX, United States; 8 Department of Mathematical Sciences, University of Texas at Dallas, Richardson, TX, United States; 9 Center for Genetics of Host Defense, University of Texas Southwestern Medical Center, Dallas, TX, United States

**Keywords:** metabolome, microbiome, multi-omics integration, predictive modeling, representation learning, supervised contrastive learning

## Abstract

Advancements in multi-omics research have demonstrated the potential of integrating human microbiome and metabolomics data to better understand physiological processes and improve prediction accuracy in studies of human health. While conventional models utilizing single-omics data provide valuable perspectives, they often fail to capture the complexity of biological systems. Recent developments in supervised contrastive learning frameworks have enhanced predictive performance for categorical responses, yet limitations persist in extending these methods to continuous outcomes. A robust model capable of addressing these gaps could significantly enhance multi-omics predictions and provide new insights into complex biological interactions. Here, we present MB-SupCon-cont, a novel supervised contrastive learning framework designed for both categorical and continuous responses in multi-omics data. MB-SupCon-cont improves prediction accuracy by incorporating a generalized contrastive loss function that defines similarity and dissimilarity for continuous responses using three distance-based weighting methods. Through simulation studies and two real-world datasets for Type 2 Diabetes (T2D) and High-Fat Diet (HFD), we demonstrate that MB-SupCon-cont consistently achieves lower prediction errors than tuned conventional models, canonical correlation analysis, and autoencoder baselines, with most reaching statistical significance. We further provide a validation-based rule for selecting the weighting method and show that the learned embeddings align more closely with the response and recover known microbe and metabolite associations. The framework also provides superior representation learning and improves data visualization in lower-dimensional spaces. These findings suggest that MB-SupCon-cont is a powerful tool for general multi-omics prediction and may have broad applicability in biomedical research.

## 1 Introduction

Integrated modeling approaches that synergistically combine multi-omics data have revolutionized biomedical research, enabling a more comprehensive understanding of complex biological relationships in human physiology. By integrating different types of biological data, researchers can explore how various systems, such as microbial communities and metabolic activities, interact within the human body. The human microbiome, a diverse collection of living microorganisms residing in a variety of body niches, plays a crucial role in disease diagnosis, prognosis, and treatments (Cho and Blaser, 2012; Human Microbiome Project C, 2012; Sender et al., 2016). Similarly, the metabolome, a complete set of small molecules involved in metabolic processes, directly impacts host physiology (Buergel et al., 2022; Nishiumi et al., 2012; Shah et al., 2012). Leveraging multi-omics data like microbiome and metabolome, researchers have developed accurate prediction models for host physiological conditions, with translational potential for improved healthcare (Claesson et al., 2017; Fromentin et al., 2022).

However, early work on prediction modeling primarily relied on single-omics data and utilized methods such as elastic net, random forests, support vector machines, and gradient boosting trees (Chen and Guestrin, 2016; Drucker et al., 1997; Zou and Zhang, 2009). While these methods offered valuable perspectives, they lacked the ability to capture the full complexity of biological systems. In contrast, integrating multi-omics data can provide deeper mechanistic insights and enhance the accuracy of prediction models. For example, in colorectal cancer, researchers have identified specific bacterial species that produce metabolites linked to an increased risk of the disease (Guthrie et al., 2017). Subsequent mechanistic studies have used multi-omics analyses to further elucidate the functions of these pathogenic species (Kang et al., 2023). Similar multi-omics approaches, especially those involving microbiome and metabolomics data, have been applied to other diseases, which demonstrates the potential of these integrative methods in a wide range of biomedical contexts (Heintz-Buschart et al., 2016; Lloyd-Price et al., 2019; Wang et al., 2020).

Existing integration models based on multivariate statistics, such as canonical correlation analysis (CCA) (Hotelling, 1936), exhibit potential, but recent advances in deep learning, including multi-view learning and particularly contrastive learning, have shown greater promise. Contrastive learning has improved image classification in computer vision and pretraining language models in natural language processing (Chen et al., 2020; Meng et al., 2021). Notably, both self-supervised contrastive learning (Chen et al., 2020) and supervised contrastive learning (Khosla et al., 2020) have been successful in image recognition. More recently, contrastive learning has also been introduced to multi-omics data analysis. Using supervised contrastive learning, MB-SupCon achieves superior performance on predicting various responses by integrating microbiome and metabolome data (Yang et al., 2022). Despite these successes, existing contrastive learning frameworks have limitations. Current supervised mechanisms only support the prediction of categorical responses, while continuous responses are quite common in biological research.

To address these challenges, we propose MB-SupCon-cont, a generalized contrastive learning framework for both categorical and continuous responses in multi-omics data. Specifically, we extend the concept of “similar and dissimilar data pairs” for continuous responses through three distance-based weighting schemes that we incorporate into a generalized contrastive loss. We have demonstrated the superiority of MB-SupCon-cont through predictions of multiple responses in both simulation studies and two real-world datasets. To make the comparison convincing, we benchmark MB-SupCon-cont against multiple classical baselines, a canonical correlation analysis method, and an autoencoder, and also provide a validation-based rule for choosing the weighting scheme. Additionally, the learned embeddings in the representation domain generate clusters that vary gradually with the response variable, which facilitates better visualization of data patterns. With these advantages, MB-SupCon-cont can serve as a general integrative prediction modeling framework in multi-omics studies and can provide versatile support to the biomedical research community.

## 2 Methods

### 2.1 Similar and dissimilar data pairs

Contrastive learning leverages the idea of learning embeddings such that similar data points are brought closer in the embedding space, while dissimilar points are pushed apart. Therefore, how to find these similar and dissimilar data pairs are critical in all types of contrastive learning methods.

In self-supervised contrastive learning, we define positive (similar) and negative (dissimilar) data pairs based on whether the data is from a paired sample collected simultaneously from the same subject. For a data pair $\left\{x_{i},y_{j}\right\};i,\;j\in \left\{1,2,\;\ldots ,\;n\right\}$ where *n* is the batch size.


$$\left\{x_{i},y_{j}\right\}=\{\begin{array}{cc} \text{positive pair} & \text{if }i=j,\\ \text{negative pair} & \text{if }i\neq j. \end{array}$$

In supervised contrastive learning for categorical response variable, positive and negative data pairs are determined based on whether the data has the same response value. Given a data pair $\left\{x_{i},y_{j}\right\}$ and their responses $\left\{r_{i},r_{j}\right\}$ where i, j∈{1,2, …, n}, the positive (or negative) pair is defined as


$$\left\{x_{i},y_{j}\right\}=\{\begin{array}{cc} \text{positive pair} & \text{if }r_{i}=r_{j},\\ \text{negative pair} & \text{if }r_{i}\neq r_{j}. \end{array}$$

However, when the response variable is continuous in a supervised contrastive learning model, the traditional definition of positive and negative data pairs becomes inapplicable, as continuous response values are rarely identical. Instead, we categorize data into similar and dissimilar pairs based on the distance between their response values. Given data points $\left\{x_{i},y_{j}\right\}$ with continuous responses $\left\{r_{i},r_{j}\right\}$, we define the distance as $d_{ij}=\left|r_{i}-r_{j}\right|$, which serves as the measure of similarity. A distance close to 0 indicates a highly similar data pair, while a larger value approaching its maximum suggests greater dissimilarity. The distance *d_ij_* is then used in the generalized contrastive loss.

### 2.2 Contrastive learning and generalized contrastive loss

The general goal of contrastive learning is to maximize the similarity of encoded embeddings in the representation domain. To measure the similarity of two vectors, the cosine similarity is the most frequent choice. From a mini-batch point of view, suppose ***x**_i_* and ***y**_j_* are different omics data vectors obtained from sample *i* and sample *j* ( i,j∈{1,2,…,n}; *n* is the batch size), respectively, *f_x_* and *f_y_* are the encoders for each omics data, then the embeddings for ***x**_i_* and ***y**_j_* are ${\text{z}}_{i}^{x}=f_{x}\left({\text{x}}_{i}\right)$ and ${\text{z}}_{j}^{y}=f_{y}\left({\text{y}}_{j}\right)$. The similarity of embeddings is defined as


$$\text{sim}\left({\text{z}}_{i}^{x},{\text{z}}_{j}^{y}\right)=\frac{{\text{z}}_{i}^{x}\cdot {\text{z}}_{j}^{y}}{\left\|{\text{z}}_{i}^{x}\right\|\left\|{\text{z}}_{j}^{y}\right\|\;}$$

where · denotes the dot product and ||·|| is the Euclidean norm of a vector.

By anchoring the embedding of one omics data ***z**^x^*, we define the generalized contrastive loss to be


$${\text{Loss}}^{x,\;y}=-E_{i}\left\{\frac{\sum_{j}w_{ij}\log P_{ij}}{\sum_{j}w_{ij}}\right\}$$

where $w_{ij}\in \left[0,1\right]$ is the weight for sample pair $\left\{{\text{x}}_{i},\;{\text{y}}_{j}\right\}$, and


$$P_{ij}=\sigma_{{\text{z}}_{i}^{x}}\left({\text{z}}_{j}^{y};\tau \right)=\frac{\text{exp}\left\{\text{sim}\left({\text{z}}_{i}^{x},{\text{z}}_{j}^{y}\right)/\tau \right\}}{\sum_{k=1}^{n}\text{exp}\left\{\text{sim}\left({\text{z}}_{i}^{x},{\text{z}}_{k}^{y}\right)/\tau \right\}}$$

is the SoftMax probability of cosine similarity of scaled embeddings and scaler $\tau \in {\mathbb{R}}_{+}$ is the temperature parameter.

By fixing the other embedding ***z**^y^* as the anchor, we will get the other piece of loss Loss*^y,x^*. For two omics data, the total contrastive loss is


$$\text{Loss}={\text{Loss}}^{x,y}+{\text{Loss}}^{y,x}.$$

Rewrite this formula in matrix form:


$${\text{Loss}}^{x,\;y}=-E\left\{\left[W\circ \log P\right]j\circ {\left[Wj\right]}^{\circ \left(-1\right)}\right\},$$

$${\text{Loss}}^{y,\;x}=-E\left\{\left[W^{T}\circ \log P^{T}\right]j\circ {\left[W^{T}j\right]}^{\circ \left(-1\right)}\right\},$$

$$\text{Loss}=-E\left\{\left(W\circ \log P\right)j\circ {\left(Wj\right)}^{\circ \left(-1\right)}+\left(W^{T}\circ \log P^{T}\right)j\circ {\left(W^{T}j\right)}^{\circ \left(-1\right)}\right\},$$

where ∘ denotes the Hadamard product (element-wise product), log ***P*** is applied elementwise, and ${\left(\cdot \right)}^{{}^{\circ}\left(-1\right)}$ denotes the Hadamard inverse. The Hadamard inverse of matrix ***X*** is defined as ${\left[X^{{}^{\circ}\left(-1\right)}\right]}_{\left(i,j\right)}=\frac{1}{x_{ij}}$. Also, vector $j_{\text{n}\times 1}={\left[1,\;1,\ldots ,1\right]}^{T}$, matrix ***W***_n×n_ is the weight matrix and matrix ***P***_n×n_ is the SoftMax probability matrix, with $P_{\left(i,j\right)}=P_{ij}$. Here, (***Wj***) holds the row sums of the weights, so its Hadamard inverse normalizes each anchor by its total weight.

By this definition, we can use one generalized formula to incorporate self-supervised contrastive loss, supervised contrastive loss on categorical responses and supervised contrastive loss on continuous responses. The key lies in the definition of the weight matrix ***W***, which captures the information of both similar and dissimilar pairs.

First, for self-supervised contrastive loss, we have $W_{\text{n}\times \text{n}}=I_{\text{n}\times \text{n}}$ where ***I*** is an identity matrix.

Second, for supervised contrastive loss on categorical responses, ***W*** is defined as


$$W_{\text{n}\times \text{n}}=\text{equal}\left[r_{\text{n}\times 1},{\left(r_{\text{n}\times 1}\right)}^{T}\right]$$

where ***r*** is the response vector and equality function equal(·) is a true/false function that checks the element-wise equalities. Equivalently,


$$W_{\left(i,j\right)}=w_{ij}=\{\begin{array}{cc} 1 & \text{if }r_{i}=r_{j},\\ 0 & \text{if }r_{i}\neq r_{j}. \end{array}$$

Third, for supervised contrastive loss on continuous responses, we propose three methods to define the weight matrix ***W***. The underlying principle is to assign a weight to a data pair based on the distance of their responses, with the weight being negatively correlated to the response distance. Namely, the larger the distance is, the smaller the weight is and vice versa.

Suppose *r_i_*, *r_j_* are the responses for a data pair $\left\{x_{i},\;y_{j}\right\}$, the norm-1 distance is $d_{ij}=\left|r_{i}-r_{j}\right|$.

Accordingly, the three weights are defined as follows.

(1) Linear weights


$$w_{ij}=1-cd_{ij},$$

where the scale


$$c=\frac{1}{\underset{i,j}{\max}\left(d_{ij}\right)+\epsilon }$$

and a small positive constant *ϵ* is to avoid zero denominator.

(2) Exponential weights


$$w_{ij}=\exp\left(-d_{ij}\right).$$

(3) Negative-log weights


$$w_{ij}^{\prime }=-\log\left(\frac{d_{ij}+\epsilon }{\underset{i,j}{\max}\left(d_{ij}\right)+\epsilon }\right),\;\;w_{ij}=\frac{w_{ij}^{\prime }}{\underset{j}{\max}\left(w_{ij}^{\prime }\right)},$$

where *ϵ* is a small positive constant to avoid zero values and we further scale $w_{ij}^{\prime }$ to contain the weights within [0,1].

In the rest of the article, we will use all three methods to train the model and compare their performance. In general, we do not expect any single method to be universally optimal for all datasets. The best method depends on the specific distribution of the response variables and the characteristics of the data. Instead of leaving this choice open, we provide a simple validation-based rule to select the weighting method and show when each method is most robust (see Results).

### 2.3 MB-SupCon-cont framework

**Figure 1:**
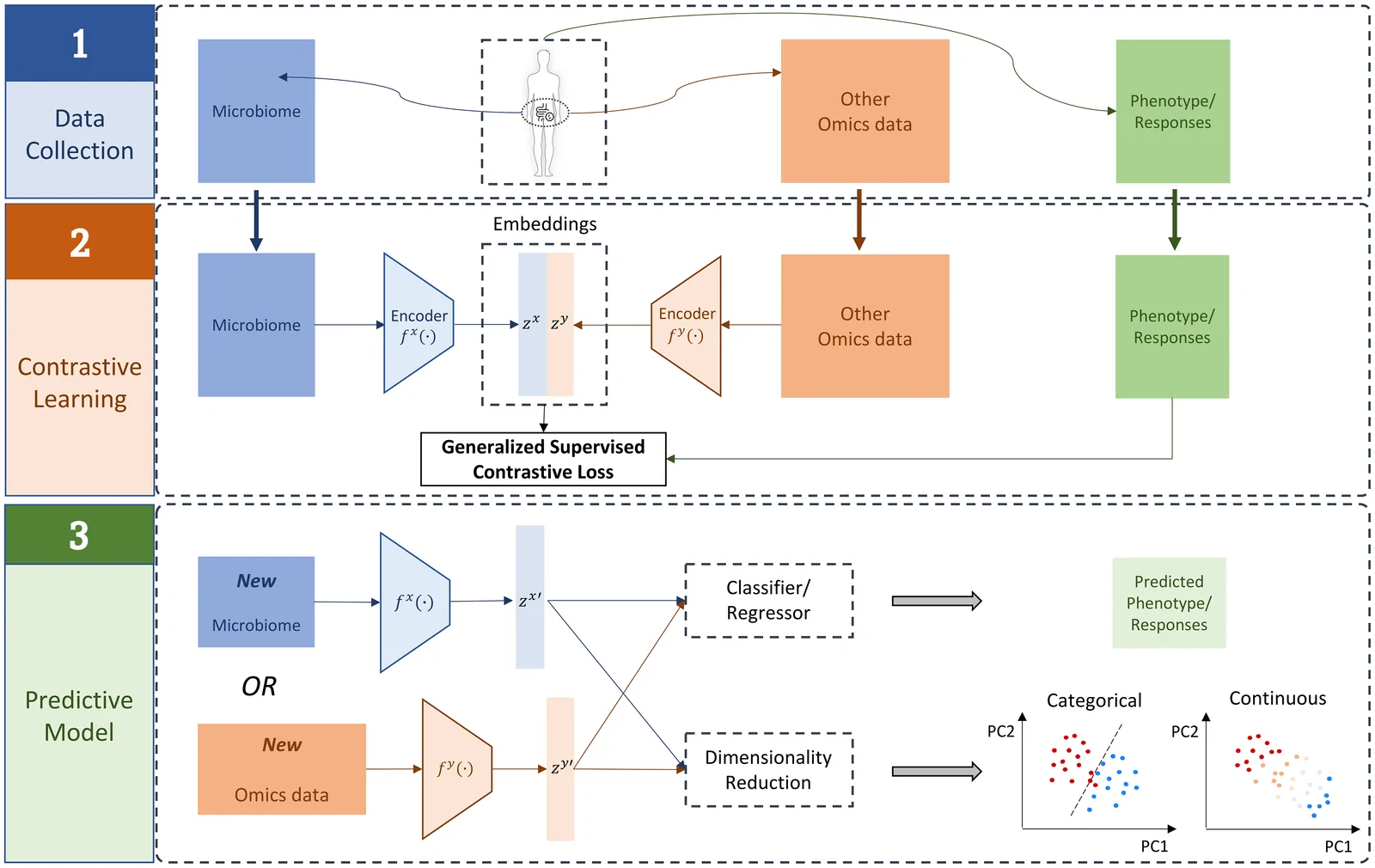
Framework of MB-SupCon-cont. Stage 1: collect paired microbiome and other related omics data, as well as phenotype information from the patients. Stage 2: train a supervised contrastive learning model using generalized contrastive loss. Stage 3: after the model is trained, given either new microbiome data or other omics data, we can improve the prediction of phenotypes and achieve better visualization.

The MB-SupCon-cont framework (**Figure 1**) is designed to integrate multi-omics data and predict both categorical and continuous responses with enhanced accuracy and interpretability. The first stage of the MB-SupCon-cont framework involves data collection. Paired omics data, such as microbiome and metabolome data, are collected along with patients’ phenotype information. In the next stage, contrastive learning is applied by training a supervised contrastive learning model to obtain encoders *f_x_* and *f_y_*. In this context, the generalized contrastive loss is used to accommodate different types of response data. In the final stage, prediction heads (either classifiers or regressors) are applied to the embeddings. When making new predictions, embeddings from either microbiome data or other omics data are selected based on their performance during the training phase. Additionally, unique trends related to the responses can be visualized in the lower-dimensional space.

### 2.4 Encoder architecture and model training

**Figure 2:**
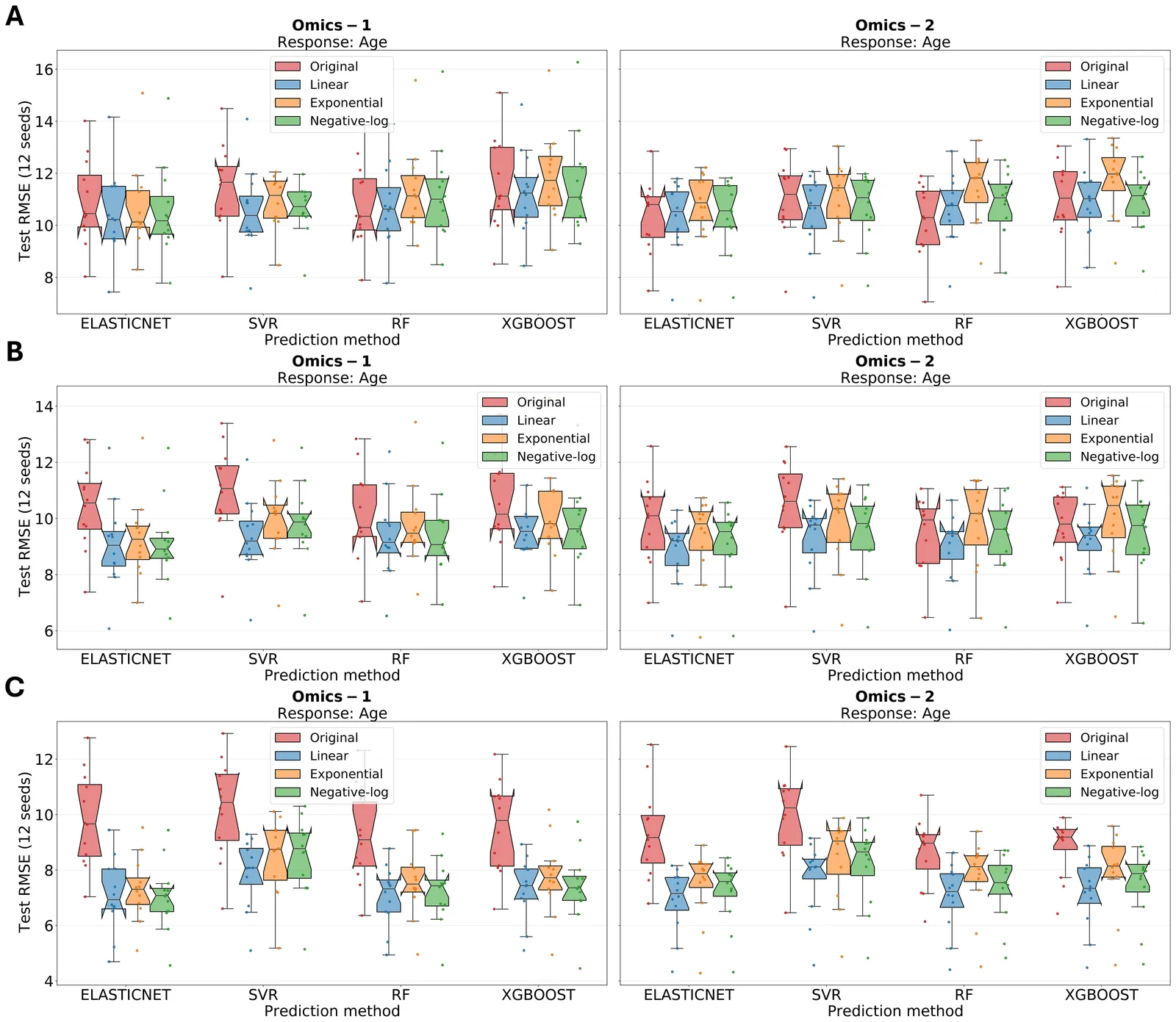
Boxplots of prediction RMSEs on testing data (15%) of simulation study from 12 random replicates when the dimension of embeddings is 20. **(A)** *µ_ρ_* = 0.4; **(B)** *µ_ρ_* = 0.6; **(C)** *µ_ρ_* = 0.8. Red boxes represent the prediction on original data. The blue, orange, and green boxes represent the prediction on embedding learning by using linear, exponential, and negative-log weighting methods.

Each omics data type has its own encoder, and the two encoders do not share weights, so each encoder can match the size and structure of its own input. Each encoder is a multilayer perceptron composed of fully connected blocks. Every block applies a linear layer, one-dimensional batch normalization, a ReLU6 activation, and dropout, followed by a final linear layer that outputs the embedding of dimension *d_e_*. For the simulation study we varied the embedding dimension over $d_{e}\in \left\{10,20,40\right\}$ to assess its effect, and we report *d_e_* = 20 as the representative setting (**Figure 2**), with *d_e_* = 10 and 40 in **Supplementary Figures 1**, **2**. For the two real datasets we used a compact embedding of *d_e_* = 10, at the low end of this range, which suits their smaller sample sizes. The hidden-layer widths of each encoder are listed in the **Supplementary Material**.

The two encoders are trained together by minimizing the generalized contrastive loss, using stochastic gradient descent with a learning rate of 0.001 and a momentum of 0.9, gradient clipping, a batch size of 32, and 1,000 epochs. Regularization is provided by dropout, batch normalization, and weight decay. For each dataset, response, and weighting scheme, the dropout rate, weight decay, and temperature were selected by the lowest validation RMSE over a grid search that used only the training and validation data. We repeated this selection across seeds and checked both how sensitive the validation RMSE was to these settings and how stable the selected values were from seed to seed. The search grid and its selected values, and the sensitivity and stability results are reported in the **Supplementary Material**.

### 2.5 Baselines and statistical testing

We compared MB-SupCon-cont against three groups of baselines, all evaluated on the same splits and with the same regression heads. For the classical baselines, we fit (1) elastic net, (2) support vector regression (SVR), (3) random forest (RF), and (4) extreme gradient boosting (XGBoost) on the original omics data. Each baseline was tuned by 5-fold cross-validation on the combined training and validation data, with the testing data held out. The hyperparameter search grids for these four baselines are given in the **Supplementary Material**. As a multi-view baseline, we fit (5) canonical correlation analysis on the training data with the same number of components as the embedding dimension and passed the canonical components of each omics data type to the regression heads. To separate the contribution of the contrastive loss from that of the neural network, we trained (6) an autoencoder for each omics data type, using the same encoder architecture but a reconstruction loss in place of the contrastive loss, and used the encoder outputs as embeddings. Its dropout and weight decay were selected on its own validation reconstruction loss, so that this baseline was tuned for the objective it optimizes.

For statistical testing, we paired the 12 test RMSE values by seed for each comparison, applied a paired Wilcoxon signed-rank test, reported a 95% confidence interval for the mean difference from a paired bootstrap with 10,000 resamples, and controlled the false discovery rate (FDR) within each table using the Benjamini-Hochberg procedure (Benjamini and Hochberg, 1995).

### 2.6 Simulation design

To benchmark the efficacy of MB-SupCon-cont, we designed a simulation study. The simulation data was generated by Reverse Principal Component Analysis (Reverse PCA), a technique we developed and detailed in the **Supplementary Material**. It reconstructed the original omics data from its lower-dimensional representation. The choice of Reverse PCA was grounded in its simplicity and ease of implementation, making it an accessible yet robust tool for our analysis. It is assumed that for two sets of omics data, a representation space exists wherein the corresponding features from both datasets are correlated. Reverse PCA is used to reconstruct a reduced-rank approximation of the original data based on their principal components. This approach guaranteed that the vital information contained within the embeddings is preserved through the reconstruction process. In this study, we assume the generated embeddings in the representation space are corresponding to the highest-variance principal components and the response variable is linked to these embeddings.

Algorithm 1 in the **Supplementary Material** gives the full implementation details of Reverse PCA. Briefly, the procedure generates two correlated omics datasets from latent embeddings and constructs a nonlinear response from linear, quadratic, and cross-omics interaction effects, and use the simulated data to evaluate MB-SupCon-cont. For each simulation setting, we generate 1,000 paired samples and set the dimensions of the two simulated omics data types to 100 and 500 features. The effect coefficients linking the latent embedding features to the response were generated from a normal distribution *N*(0,25), and the generated response is scaled to 0 to 100 assuming it contains “Age” information. We also considered several values for the average feature correlation, $\mu_{\rho }\in \left\{0.4,\;0.6,\;0.8\right\}$, and the embedding dimension, $d_{e}\in \left\{10,20,40\right\}$.

To test whether the advantage of MB-SupCon-cont holds beyond the Reverse PCA design, we added three more realistic regimes to the simulation. In the first regime, only a subset of the representation carries signal, and the remaining components are uninformative noise. In the second regime, only one omics layer is informative. In the third regime, the response coefficients are sparse. As a further check on the realism of the design, we validated Reverse PCA on the two real datasets by comparing the original data with their low-rank reconstruction and measuring the fraction of feature variance that the reconstruction recovered. We describe the regimes and the reverse PCA validation in full in the **Supplementary Material**.

## 3 Results

### 3.1 Simulation study based on Reverse PCA

To showcase the efficacy and benefits of MB-SupCon-cont, the prediction on simulated original data is compared to the prediction on learned embeddings. Our simulation demonstrated the superiority of MB-SupCon-cont in predicting continuous responses. Specifically, as the average correlation of embedding features rises, the advantage of MB-SupCon-cont grows correspondingly. At $\mu_{\rho }=0.8$, the benefit of using MB-SupCon-cont is most notable. As shown in **Figure 2**, in most scenarios the blue, orange and green boxes (prediction on embeddings) are significantly lower than the red box (prediction on original data). Additionally, the linear weighting method performs marginally better than the other two when predicting the simulated “Age” variable. This illustrates that MB-SupCon-cont can enhance prediction performance based on embeddings, and this enhancement increases with the average correlation coefficient of embeddings (**Figure 2**; **Supplementary Figures 1**, **2**).

We repeated the simulation under the three more realistic regimes described in the Methods, which add uninformative noise components, make only one omics layer informative, or make the response coefficients sparse. In the single informative omics and sparse regimes, the embeddings still gave lower prediction error than the original data, and when only one omics layer carried signal, the informative layer produced the better predictions, as expected. In the noise regime, where most of the representation is uninformative, the advantage of the embeddings shrank to roughly a tie at the best head, as expected when there is little shared structure to exploit. This shows that the advantage of MB-SupCon-cont holds whenever the two omics layers share response-related signal and does not depend on the specific structure of the Reverse PCA design. The full results for all regimes are given in the **Supplementary Material** (**Supplementary Figure 6**; **Supplementary Table 4**).

We also assessed how well Reverse PCA reproduces the real data. At an embedding dimension of 20, the low-rank reconstruction recovered about 79% of the feature variance for the T2D microbiome, 49% for the T2D metabolome, 34% for the HFD microbiome, and 46% for the HFD metabolome, which shows that Reverse PCA is a reasonable generative model for real omics data. The features with the poorest reconstruction were likely to be low-abundance taxa and metabolites, which may be harder for principal component analysis (PCA) to capture accurately.

### 3.2 Application – type 2 diabetes study

We benchmarked MB-SupCon-cont to paired microbiome and metabolome data (Zhou et al., 2019). This study enrolled 720 samples of prediabetes patients. Three continuous responses that are all associated with the disease status of diabetes patients were considered for prediction, which are Age, Body Mass Index (BMI) and Steady State Plasma Glucose (SSPG) level.

To assess the prediction performance, we employed the Root Mean Square Error (RMSE) as the evaluation metric. For a comparative perspective, predictions were also made on the original microbiome and metabolome data. The MB-SupCon-cont results used the best hyperparameter combination selected on the validation data, and the baselines were also tuned, as described in the Methods. The dataset was partitioned into a training set (70%), a validation set (15%), and a testing set (15%). The dimensions of the microbiome data are 720 × 96, and for the metabolome data, they are 720 × 724. We executed the analysis 12 times, each with a unique random seed for the training-validation-testing splits and recorded the RMSE for all prediction methods during each run.

**Figure 3:**
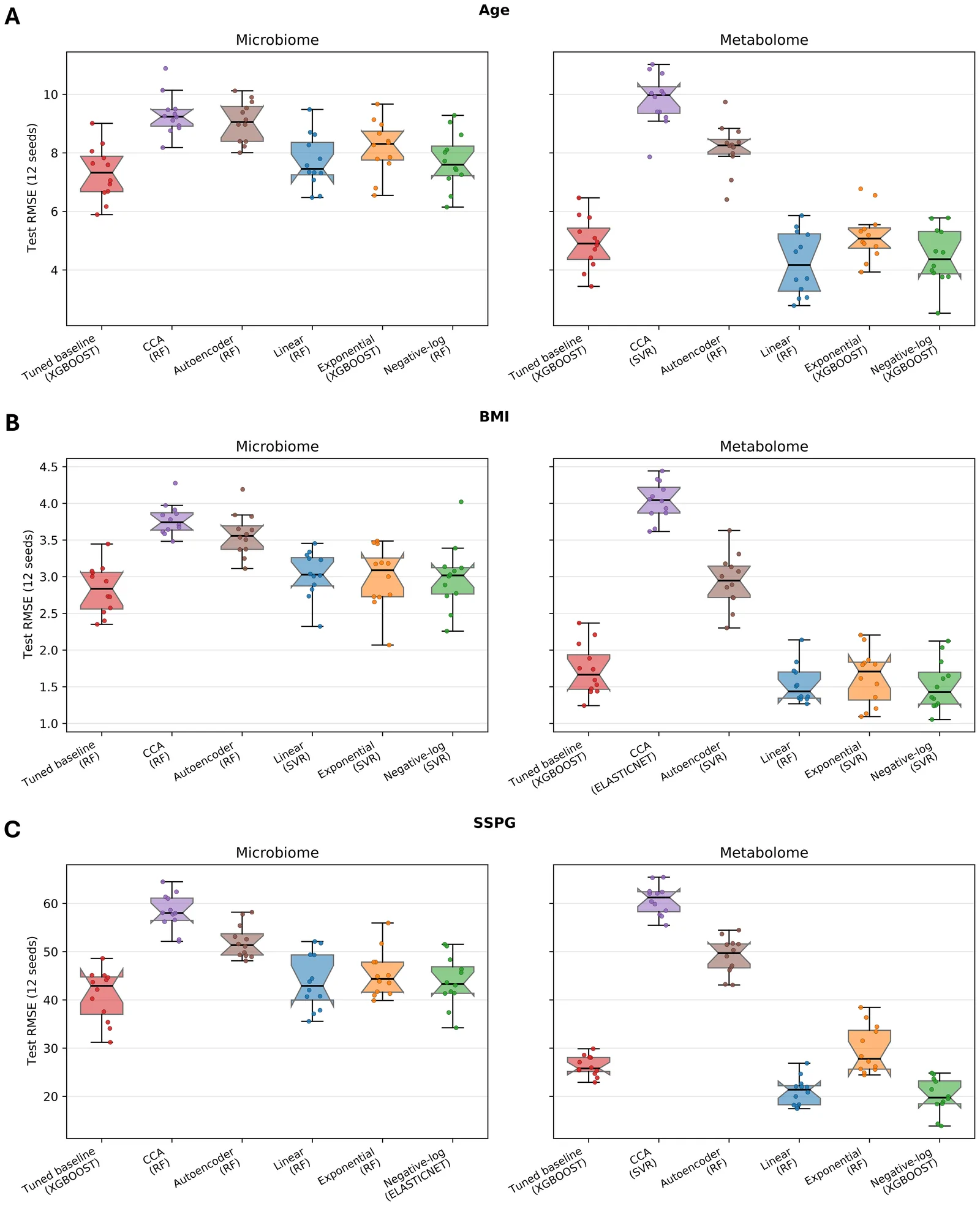
Boxplots of prediction RMSEs on testing data from 12 random replicates, by using different methods for three continuous responses (T2D study). **(A)** Age; **(B)** BMI; **(C)** SSPG. Each method is shown at its best-performing prediction head, given in parentheses below the box. Boxes are notched at the median and the points are the 12 per-seed RMSEs. Colors: red, tuned baseline; purple, canonical correlation analysis; brown, autoencoder; blue, linear; orange, exponential; green, negative-log.

In **Table 1**, the mean RMSE values averaged over the 12 runs are contrasted across the various prediction methods. For predictions based on MB-SupCon-cont embeddings, each weighting method is shown with its best performing prediction head, and the full per-head results are given in the **Supplementary Material**. Predictions based on metabolome embeddings achieved the lowest RMSE for all three responses. The best average test RMSE for Age was achieved with linear weights (4.2379), for BMI with negative-log weights (1.5221), and for SSPG with negative-log weights (20.0849). Each of these was significantly more accurate than the best tuned baseline (paired Wilcoxon signed-rank test, *p* = 0.0005 for Age, *p* = 0.0093 for BMI, and *p* = 0.0010 for SSPG; FDR-adjusted p-value of at most 0.014). The canonical correlation analysis and autoencoder baselines were clearly worse (FDR-adjusted p-value of at most 0.004). The autoencoder uses the same encoder architecture, was tuned on its own reconstruction objective, and differs from MB-SupCon-cont only in the loss function, so this shows that the improvement comes from the contrastive learning formulation rather than from the neural network architecture alone. For the microbiome data, the tuned classical baselines had lower average RMSE than MB-SupCon-cont for all three responses (with only BMI significant; FDR-adjusted *p* = 0.023), which indicates that in this study the metabolome carries the stronger integrative signal. A boxplot comparison of all prediction methods for predicting three responses is also presented in **Figure 3**.

**Table 1:** Prediction error in the T2D study.

Response	Tuned baseline	CCA	Autoencoder	MB-SupCon-cont		
				*Linear*	*Exponential*	*Negative-log*
Microbiome						
Age	7.3189 (XGBoost)	9.3040 (RF)	9.0645 (RF)	7.7085 (RF)	8.1742 (XGBoost)	7.7273 (RF)
BMI	2.8278 (RF)	3.7786 (RF)	3.5725 (RF)	3.0339 (SVR)	2.9902 (SVR)	2.9928 (SVR)
SSPG	40.9987 (XGBoost)	58.2669 (RF)	52.0979 (RF)	43.7351 (RF)	45.3779 (RF)	43.8193 (ElasticNet)
Metabolome						
Age	4.9137 (XGBoost)	9.8050 (SVR)	8.1722 (RF)	**4.2379** **(RF)**	5.1786 (XGBoost)	4.4586 (XGBoost)
BMI	1.7291 (XGBoost)	4.0323 (ElasticNet)	2.9412 (SVR)	1.5360 (RF)	1.6323 (SVR)	**1.5221** **(SVR)**
SSPG	26.2918 (XGBoost)	60.7627 (SVR)	49.0548 (RF)	21.0723 (RF)	29.7155 (RF)	**20.0849** **(XGBoost)**

Average RMSEs on testing data for predicting Age, BMI and SSPG over 12 random splits. Each method is shown at its best-performing prediction head, given in parentheses. For each response, the lowest RMSE across all methods and omics data types is highlighted in bold.

**Figure 4:**
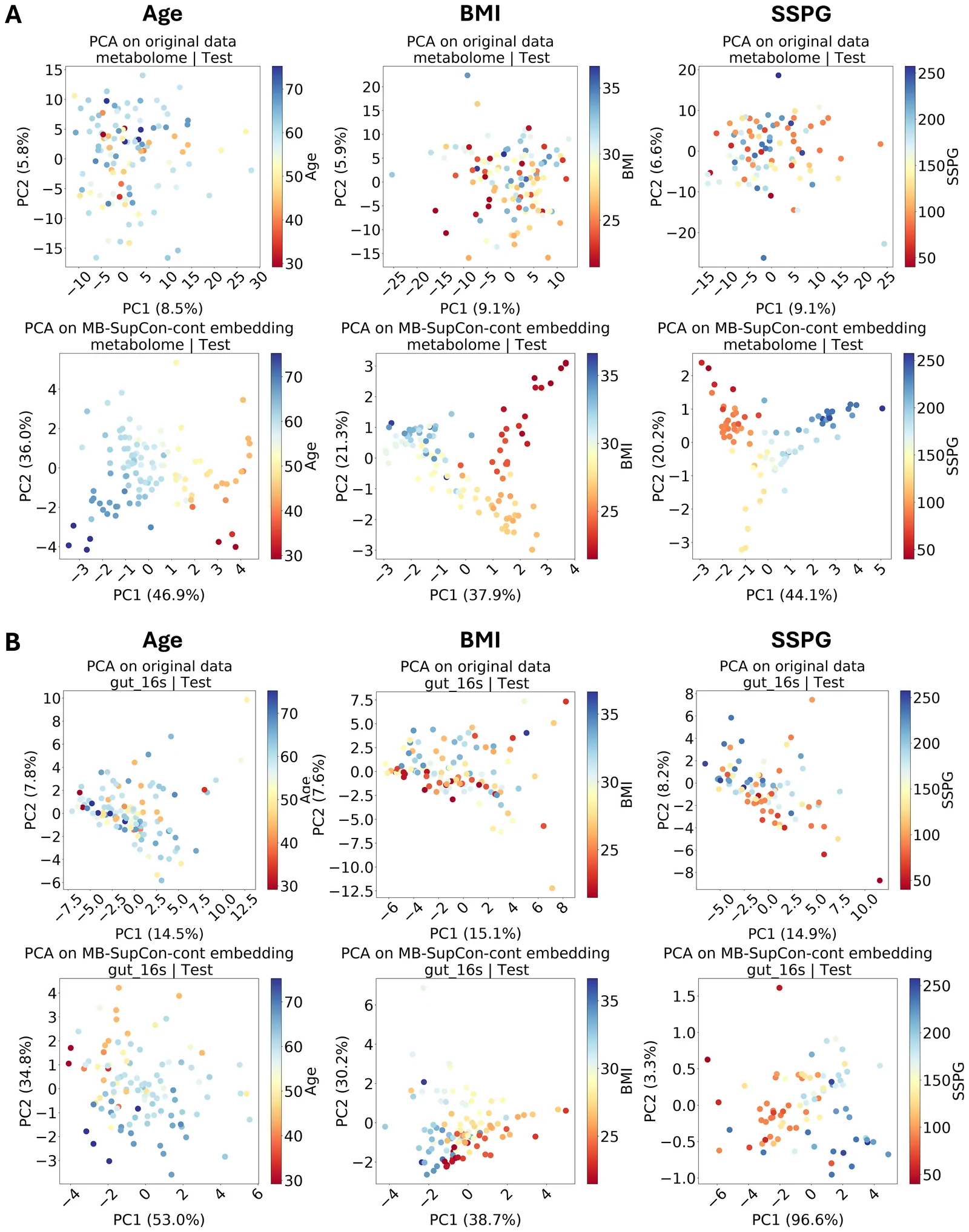
PCA scatter plots (PC2 vs. PC1) on testing data for responses Age, BMI, and SSPG (T2D study). **(A)** shows the lower-dimensional PCA projections of the original metabolome data (first row) and the MB-SupCon-cont metabolome embeddings (second row). **(B)** presents the lower-dimensional PCA projections of the original microbiome data (first row) and the MB-SupCon-cont microbiome embeddings (second row). Each embedding is shown at the weighting method that performed best for that omics and response. The percentage on each axis is the proportion of variance explained by that principal component in PCA.

For comparison, PCA was performed on both (1) the original microbiome and metabolome data, and (2) the MB-SupCon-cont microbiome and metabolome embeddings, each shown at the weighting method that performed best for that omics and response. From **Figure 4**, both Panel A (metabolome) and Panel B (microbiome) show that the MB-SupCon-cont embeddings (second rows) exhibit a much more distinctive varying trend in the lower-dimensional space compared to the original metabolome and microbiome data (first rows). This indicates that MB-SupCon-cont embeddings provide more accurate response-related representations of the omics data and enhance the visualization of underlying data patterns. The first two principal components of the metabolome embeddings explain a large proportion of the variance (about 0.83 for Age, 0.59 for BMI, and 0.64 for SSPG), compared with about 0.14 to 0.16 for the original metabolome data.

### 3.3 Application – high-fat diet study

To further validate the superiority of MB-SupCon-cont in predicting continuous responses, we applied it to another independent multi-omics murine model studying the effect of a high-fat diet on the microbial and metabolic changes in mice (Morton et al., 2019). This model utilized 434 paired microbiome and metabolome data for integration. In contrast to the previous T2D study, the HFD study presented substantially higher dimensionality in the paired omics data, with the microbiome data dimension being 434 × 913 and the metabolome data dimension being 434 × 11,978. We still split the dataset into training (70%), validation (15%), and testing data (15%). The analysis was conducted 12 times using different random seeds. As in the T2D study, the MB-SupCon-cont results used the best hyperparameter combination selected on the validation data, and the baselines were also tuned. Responses Age and Weight are selected as the prediction tasks.

**Supplementary Table 1** and **Supplementary Figure 3** have shown the comparison of prediction RMSEs on testing data (15% of all data) across different methods. Similar to the T2D study, predictions based on metabolome embeddings achieved the lowest average RMSE for both Age and Weight. The best average test RMSE for Age was achieved with negative-log weights (7.0759) and for Weight with linear weights (6.3413). For Age, this was significantly lower than the best tuned baseline (paired Wilcoxon signed-rank test, *p* = 0.0005; FDR-adjusted p-value is 0.0010), while for Weight the improvement was small and not statistically significant (*p* = 0.3804). The canonical correlation analysis and autoencoder baselines were again clearly worse for both responses (FDR-adjusted p-value of at most 0.0010). Meanwhile, the color variations corresponding to different response values are clearly distinguishable in the lower-dimensional PCA scatter plots (PC2 vs. PC1) of metabolome and microbiome embeddings (**Supplementary Figures 4**, **5**). The first two principal components of these embeddings explain a large share of their variance (about 0.98 for Weight and 0.52 for Age in the metabolome embeddings, and about 0.97 for Weight and 1.00 for Age in the microbiome embeddings), compared with about 0.16 for the original metabolome data and 0.09 for the original microbiome data. This study reaffirms the stable and consistent performance of MB-SupCon-cont.

### 3.4 Weighting-scheme selection and robustness to response noise

We defined three weighting schemes, which raises two questions: how to choose among them, and whether all schemes are worth keeping. Our results show that the best scheme depends on the response and can even differ between the two omics. In the T2D study, the lowest error on the metabolome came from linear weights for Age and negative-log weights for BMI and SSPG, while on the microbiome it came from linear weights for Age and SSPG and exponential weights for BMI. In the HFD study, the lowest error on the metabolome came from negative-log weights for Age and linear weights for Weight, while on the microbiome linear weights were best for both Age and Weight (**Table 1** and **Supplementary Table 1**). Thus, the best scheme depends on the response and should be selected from the data rather than fixed in advance.

To make this choice automatic, we select the scheme by validation performance. For each dataset and response, we take the three schemes at their best prediction heads and keep the one with the lowest validation RMSE. We then evaluate the selected scheme on the held-out test data. This rule selected the best performing scheme on the test set in nine of the ten (90%) scenarios across the two studies. In the one exception, HFD Weight, the scheme it chose was only about 0.2 RMSE behind the best. The validation-based rule is therefore a simple and dependable way to make the choice.

**Figure 5:**
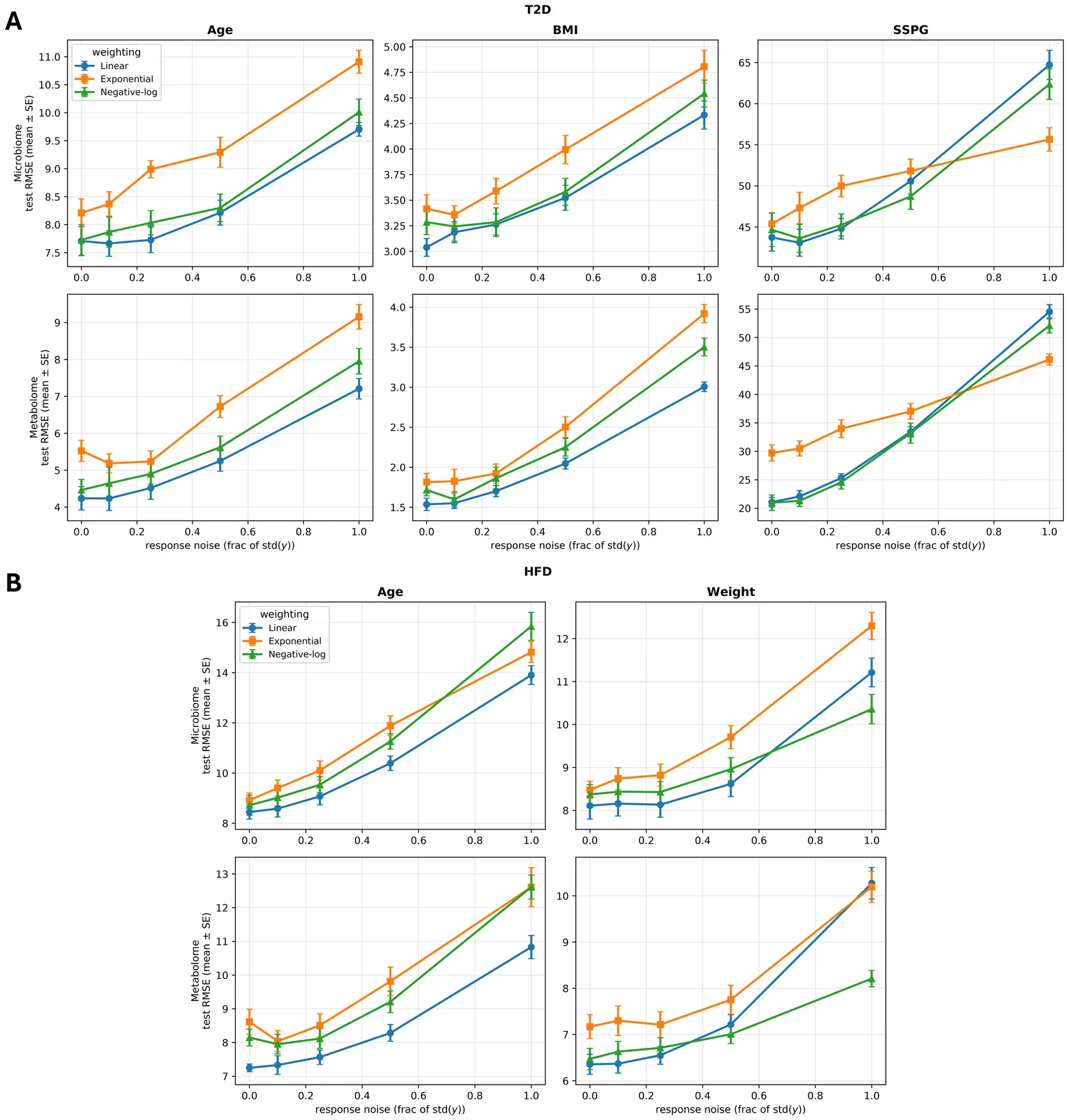
Robustness to response noise of the three weighting schemes. Test RMSE of the linear, exponential, and negative-log schemes as the level of response noise added to the training and validation responses increases, while the test response is kept clean. For this comparison the schemes are evaluated at a fixed random forest head, so that only the weighting scheme varies. **(A)** T2D. **(B)** HFD. Within each panel the rows are the two omics data types (first row microbiome, second row metabolome) and the columns are the responses. Points are means over the 12 splits and the error bars are one standard error.

The schemes also differ in how they tolerate measurement noise in the response. To assess their robustness, we added Gaussian noise to the training and validation responses at levels ranging from 0 up to 1 standard deviation (SD) of the response (a noise fraction of 0 to 1), while keeping the test response clean. As shown in **Figure 5**, the three schemes degraded at different rates as noise increased, and thus their relative ranking could change depending on response ranges. For responses with a narrow range, such as T2D Age and BMI, the ranking of the three schemes remained largely unchanged as the noise increased. For responses with a wide range, such as T2D SSPG and HFD Weight, the ranking changed under heavy noise. Also, the exponential and negative-log weighting schemes became the most robust under heavier noise because they placed more weight on pairs with similar responses. For example, on the metabolome embeddings with the prediction head fixed at random forest, we varied the weighting scheme and the noise level (**Figure 5**). Measured by the increase in mean test RMSE as the response noise rose from 0 to 1 SD, the exponential scheme was the most robust for T2D SSPG, with its error rising by only 16.4 compared with 33.5 for linear weights. For HFD Weight, the negative-log scheme was the most robust, with its error increasing 1.7 compared with 3.9 for linear weights. In both cases, at the highest noise level, the more robust scheme also had the lowest validation RMSE and was therefore selected by the validation-based rule.

These results lead to a simple guideline. The weighting scheme is not a fixed part of the method but a data-driven choice that the validation-based rule can make reliably. The exponential and negative-log schemes earn their place because they are more robust than linear weights when a wide-range response may be measured with error. The three schemes therefore form a useful family rather than redundant options.

### 3.5 MB-SupCon-cont embedding geometry reflects response structure

The principal component scatter plots suggest that the MB-SupCon-cont embeddings organize samples by the response. To test this directly, we measured how closely the geometry of the embedding space matches the geometry of the response. For each embedding we computed the pairwise Euclidean distances between samples, and did the same for the response, then measured the correlation between the two distance matrices with a Mantel test (Mantel, 1967) using 999 permutations. A high Mantel correlation means that samples close in the embedding space also have similar responses.

**Figure 6:**
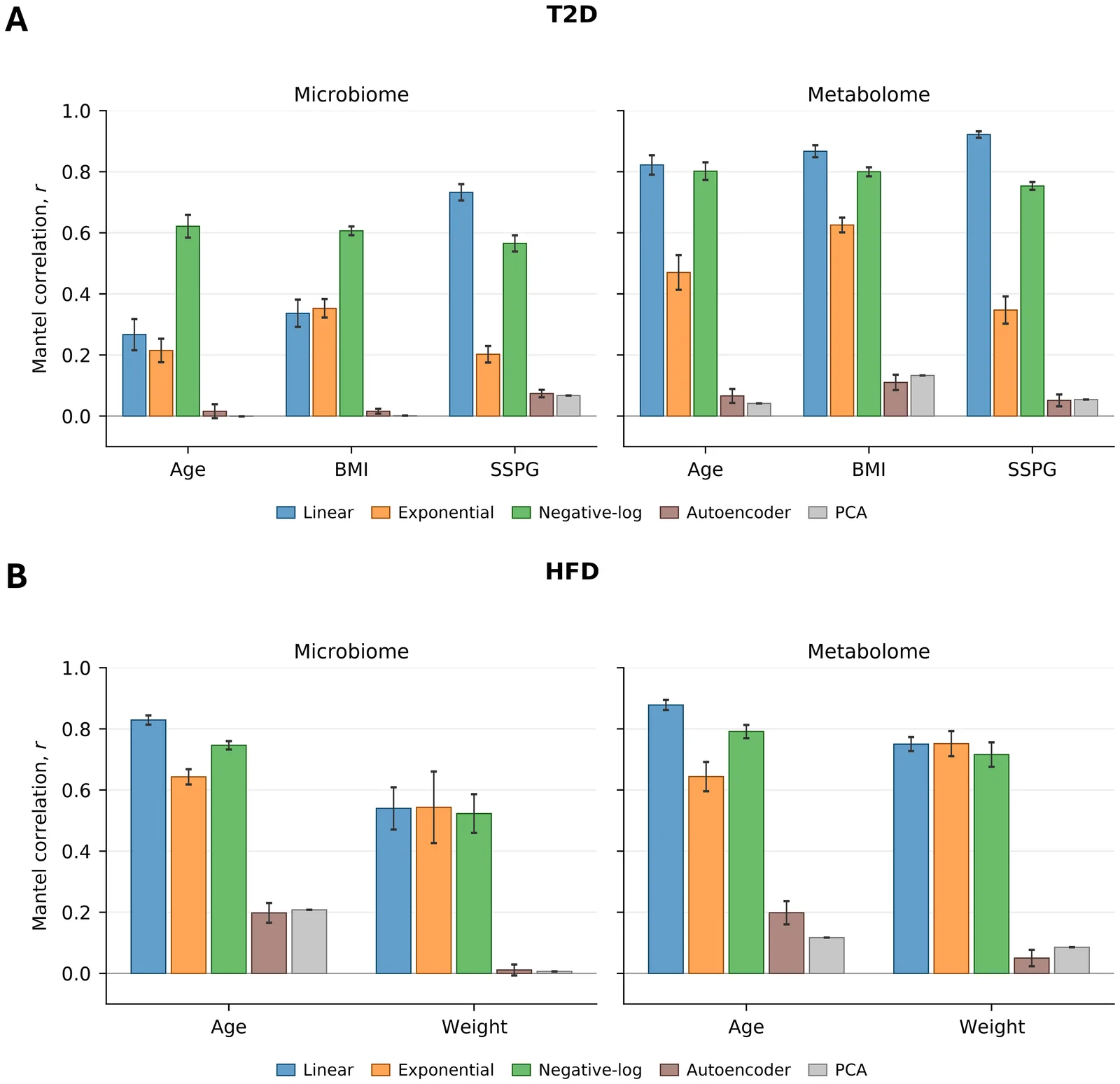
Mantel correlation between embedding distances and response distances for MB-SupCon-cont with the three weighting schemes (linear, exponential, negative-log), the autoencoder, and PCA of the original data. **(A)** T2D. **(B)** HFD. Within each panel the two subplots are the microbiome and metabolome embeddings, and the groups along the horizontal axis are the responses. Bars are the mean over the 12 splits, and the error bars show one standard deviation. Higher bars mean the embedding geometry aligns more closely with the response geometry.

The Mantel test quantitatively confirms that distances in the best-scheme embeddings track the response distances much more closely than distances in the original data do. In the T2D study the Mantel correlations were about 0.27 to 0.82 across the two omics and three responses, and in the HFD study about 0.54 to 0.83 across the two omics and both responses (all significant with *p* = 0.001), compared with about 0.00 to 0.21 for PCA of the original data. For the autoencoder, although tuned on its own reconstruction objective, it reached only about 0.01 to 0.20 and was significant in only four of the ten cases. This confirms that the MB-SupCon-cont embeddings encode response structure that neither linear nor deep unsupervised representation recovers (**Figure 6**; **Supplementary Table 11**).

We also measured the effective dimensionality of each embedding using the participation ratio of its singular value spectrum, where a low value means the embedding is concentrated in a few directions and a high value means it is more spread out. The three weighting schemes produced clearly different geometries. The linear scheme gave the most concentrated embeddings, with a participation ratio around 1 to 2, while the exponential and negative-log schemes gave more spread-out embeddings, with a participation ratio up to about 7. The linear scheme was strongest on most metabolome cells (Mantel correlation about 0.82 to 0.92 on the T2D metabolome), while the negative-log scheme was strongest on some microbiome cells, such as the T2D microbiome for Age and BMI (about 0.62 and 0.61, compared with about 0.27 and 0.34 for linear weights). These results show that the weighting schemes produce genuinely different embedding geometries, not just minor variations, which gives a geometric explanation for why different schemes can be preferred on different data.

### 3.6 The embeddings encode interpretable microbe and metabolite associations

As MB-SupCon-cont embeddings are supervised by the response, we next examined which microbes and metabolites they encode. Features were ranked by their association with the response using both the embedding and the raw data, and the resulting two rankings were compared. For the first ranking, we define the response axis as the direction in the embedding space along which the response changes fastest, obtained by regressing the response on the embedding and keeping the fitted values. The regression is fitted on the training samples and applied to the testing samples, so the axis is never evaluated on the samples that defined it. Every feature is then ranked by its Spearman correlation with this axis. The second ranking comes from the raw data, where every feature is ranked by its Spearman correlation with the response itself. Both rankings use the testing samples only and are averaged over the 12 splits. **Table 2** reports the top five microbes along each response axis, with both correlations and the number of splits in which each feature recurs. The full ranked lists for both microbes and metabolites are given in **Supplementary Table 12**.

**Table 2:** Leading microbes along each response axis.

Study	Response	Top microbes (axis, direct, splits)	
T2D	Age	genus Blautia (+0.27, +0.24, 9/12)	genus Parabacteroides (+0.25, +0.15, 8/12)
		family Lachnospiraceae (+0.22, +0.16, 7/12)	genus Clostridium IV (+0.22, +0.21, 8/12)
		genus Prevotella (-0.20, -0.10, 7/12)	
T2D	BMI	genus Odoribacter (-0.29, -0.22, 11/12)	genus Barnesiella (-0.28, -0.27, 11/12)
		genus Clostridium XlVa (+0.26, +0.21, 9/12)	family Streptococcaceae (+0.21, +0.15, 6/12)
		unclassified Bacteria (-0.20, -0.13, 6/12)	
T2D	SSPG	unclassified Clostridiales (-0.49, -0.46, 12/12)	unclassified Bacteria (-0.48, -0.37, 12/12)
		unclassified Lachnospiraceae (-0.38, -0.29, 10/12)	family Clostridiaceae 1 (-0.37, -0.37, 10/12)
		genus Clostridium sensu stricto (-0.37, -0.37, 11/12)	
HFD	Age	family Muribaculaceae, S24-7 (-0.45, -0.43, 8/12)	genus Anaeroplasma (-0.42, -0.38, 8/12)
		genus Bacteroides (-0.39, -0.36, 5/12)	order Rickettsiales (-0.38, -0.38, 6/12)
		unclassified Bacteria (-0.38, -0.37, 5/12)	
HFD	Weight	family Lachnospiraceae (+0.53, +0.45, 9/12)	genus Candidatus Arthromitus (-0.49, -0.42, 8/12)
		family Muribaculaceae, S24-7 (-0.49, -0.47, 6/12)	species Ruminococcus gnavus (+0.49, +0.35, 6/12)
		family Erysipelotrichaceae (+0.48, +0.45, 7/12)	

For each response, the five microbes most strongly correlated with MB-SupCon-cont response axis at the best weighting method for each omics data type, are shown in rank order from left to right. The axis is fitted on the training samples and applied to the testing samples, where both correlations are computed and averaged over the 12 splits. In parentheses, each entry gives the correlation with the axis, the direct correlation with the response, and the number of splits in which the microbe entered the top 20.

**Figure 7:**
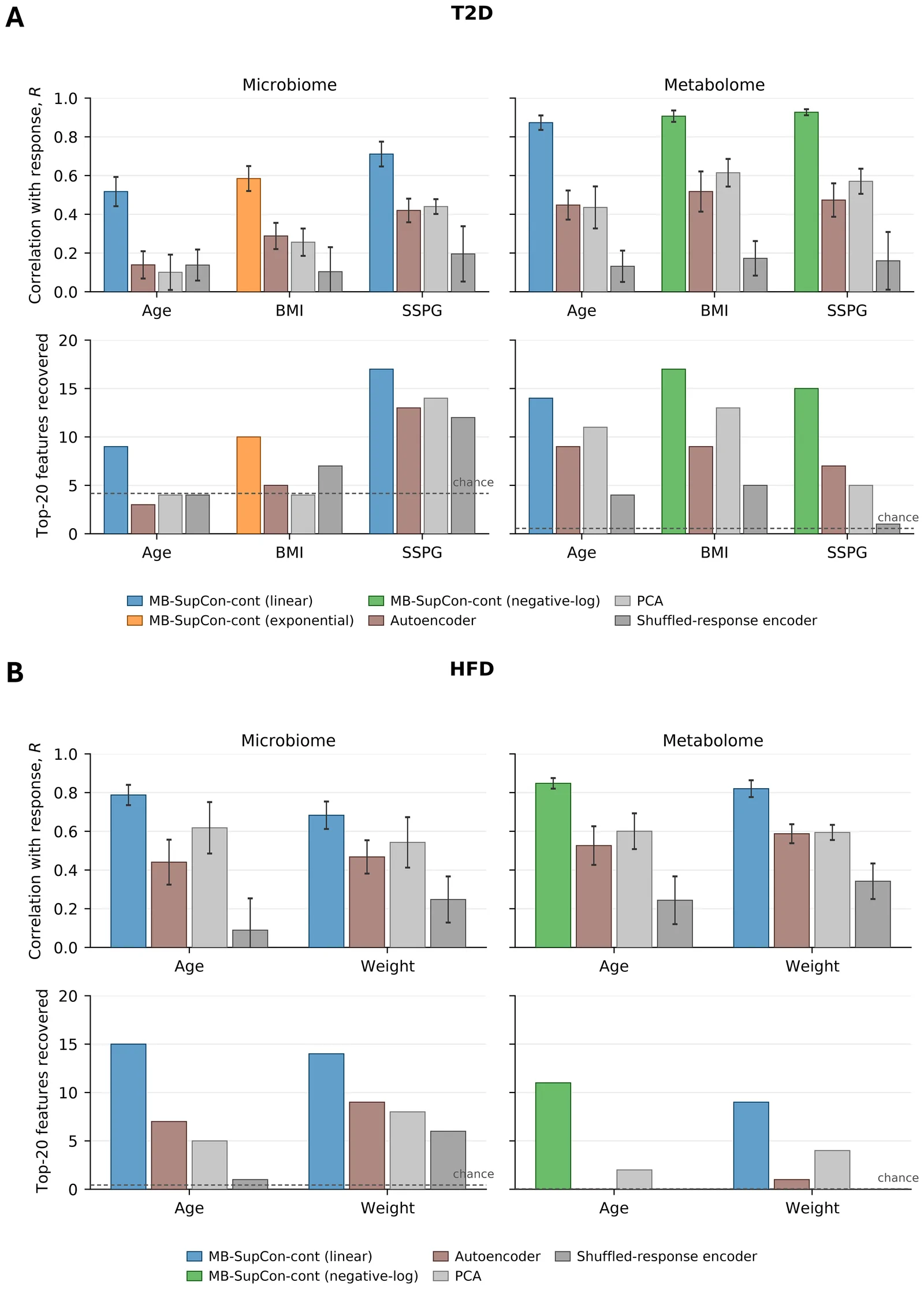
Recovery of the response and its associated features by the MB-SupCon-cont embeddings and three controls. **(A)** T2D. **(B)** HFD. Within each panel the left and right subplots are the microbiome and metabolome embeddings, and the groups along the horizontal axis are the responses. The four bars are MB-SupCon-cont at the best weighting method for that cell, the autoencoder, PCA of the raw data, and the shuffled-response encoder. All four were passed through the same response-axis pipeline on the same held-out samples. Upper row: the correlation between the held-out response and the response axis, with bars the mean over the 12 splits and error bars one standard deviation. Lower row: how many of the 20 directly associated features the axis recovers, with the dashed line the overlap expected by chance (hypergeometric mean). Colors: blue, linear; orange, exponential; green, negative-log; brown, autoencoder; light grey, principal component analysis; dark grey, shuffled-response encoder.

The two rankings largely agree. The response axis follows the held-out response with a correlation of 0.52 to 0.93 across the studies, omics data types, and responses, and the two rankings share 9 to 17 of their top 20 features, against 0.03 to 4.2 expected by chance. Three controls were passed through the same pipeline on the same samples: the same encoder retrained on permuted responses (shuffled-response encoder), an autoencoder, and principal component analysis of the raw data. The shuffled-response encoder reached a correlation of at most 0.34 and shared at most 12 of the top 20 features, and the two unsupervised representations reached at most 0.62 and 14 of 20. MB-SupCon-cont was ahead of all three in every one of the ten cases (**Figure 7**; **Supplementary Table 13**). The shuffled-response encoder is the informative comparison, because it differs from MB-SupCon-cont only in that its supervision has been destroyed. The biological signal captured by the embedding is driven by the response and not from the omics data alone. The leading features also recur across splits, and the strongest of them entered the top 20 in all 12. For these features, the correlation with the axis exceeded the direct correlation in every case, which indicates that the embedding retains the response signal they carry rather than diluting it.

The leading features are also consistent with known biology. In the T2D study, the metabolite leading the Age axis was dehydroepiandrosterone sulfate (DHEA-S), matching its well-established decline over the human lifespan (Orentreich et al., 1984). The BMI axis was led by Odoribacter, a butyrate-producing genus, which decreased as BMI increased (Gomez-Arango et al., 2016). Along the SSPG axis, a measure of insulin resistance, unclassified Clostridiales taxa decreased, which agrees with a report from the same prediabetes cohort that linked a decline in the order Clostridiales to lower insulin sensitivity (Zhou et al., 2020). In the HFD study, members of the family Muribaculaceae (S24-7) decreased along the Weight axis while Erysipelotrichaceae increased, in agreement with previous reports on diet-induced changes in the mouse gut microbiome (Daniel et al., 2014; Fleissner et al., 2010). On the metabolome side of the same axis most of the leading features were never identified, and the highest ranked compounds that do carry an identification were the bile acids glycodeoxycholic acid and chenodeoxycholic acid, both of which increased with Weight, which is consistent with the evidence that high-fat diets can alter host bile acid composition (Devkota et al., 2012).

These results show that MB-SupCon-cont embeddings are built from biologically meaningful features and can still be read in terms of individual microbes and metabolites. As the encoder is supervised by the response, they recover the known response biology. The embeddings are therefore interpretable, and they can be read as a summary of the microbes and metabolites that carry the response signal.

## 4 Discussion

We develop a novel generalized contrastive learning framework with a newly designed weighting scheme that extends the traditional supervised contrastive learning approach. It can integrate multi-omics datasets by deriving omic-specific embeddings and achieve four advantages: (1) Proposed a unified contrastive loss: by defining similar and dissimilar pairs from the distance between response values, one loss covers the self-supervised, categorical supervised, and continuous supervised cases, which opens the supervised framework to the continuous responses common in biological research. (2) Enhanced predictive performance (lower RMSE): the model demonstrates superior prediction performance, enabling more accurate and reliable analysis of multi-omics data. (3) Advanced representation learning: employing embeddings as a means of representation learning facilitates the extraction of meaningful and comprehensive information from the omics data. Samples close together in the embedding have similar responses, and this structure is built from the microbes and metabolites directly associated with the response, which adds biological interpretability. (4) Enhanced visualization of data patterns on lower-dimensional space: the model yields enhanced visualization by revealing a smooth yet distinguishable color gradient in PCA scatter plots for continuous variables. We have demonstrated its performance through extensive simulation and real data analysis. These improvements hold against several tuned baselines, canonical correlation analysis, and an autoencoder, and are statistically significant.

The three weighting methods deserve comment, as they proved neither interchangeable nor reducible to a default. Once each method is tuned on its own, the best one depends on the response and can differ between the two omics data types for the same response. No simple property of the response, such as its range, can by itself anticipate which will win. The embedding geometry suggests why. The methods differ in how sharply they discount distant pairs, which determines how strongly they collapse the embedding onto the response. The degree of collapse that suits a dataset is a property of that dataset and cannot be known beforehand. This is why we prefer a validation-based rule to a recommendation. The rule uses no test data and costs nothing beyond the training the model already requires. It also adapts to noise in the response. When a wide-range response is measured with error, the exponential and negative-log methods degrade more slowly than linear weights, and they also win on validation RMSE under that noise. The rule therefore selects them without being told that the response is noisy. This robustness matters in practice, as measurement error is common for some phenotypes such as SSPG. The three methods are best regarded as a family whose selection belongs to fitting the model, not as a choice left to the user.

Given these advantages and the outstanding performance of MB-SupCon (Yang et al., 2022) and MB-SupCon-cont, another important question arises: which datasets are worth integrating in the first place? It is clear that not every pair of datasets can be effectively integrated. A contrastive model learns from the contrast between the two datasets, so it gains information only when the two are related enough to share signal, yet different enough that each one contributes something the other does not. This is the idea of “informativeness” (Yeh et al., 2022). An informative pair of datasets should neither be “easy positives” (too similar to offer more information than a single dataset) nor “easy negatives” (too unrelated that they provide no meaningful integration). In biological studies, this means selecting datasets that are biologically connected yet offer distinct and complementary insights into the system under study. Our results show that this balance matters in practice. In both studies, the metabolome embeddings carried the stronger integrative signal, while the tuned baselines remained competitive on the microbiome data. The benefit of integration therefore depends on which omics layer carries the response-related signal, and it should be assessed for each dataset rather than assumed.

In addition, while we demonstrated the utilities of MB-SupCon-cont in integrative microbiome and metabolomics datasets, this work can be extended to other omics types. Nothing in the framework is specific to these two data types, as the encoders are independent and sized to their own input, and the loss sees only the responses and the two sets of embeddings. For example, integrating host genetics, genomics, epigenetics, and proteomics, along with microbiome and metabolomics is also feasible. Applying the framework to a new pairing would require its own preprocessing, tuning, and biological interpretation, and we leave this to future work. Our proposed framework nevertheless has the potential to improve integrative data analysis in broader settings.

We also note that both datasets are of modest size (720 and 434 samples), which is small for deep learning. One reason MB-SupCon-cont remains effective at this scale is that the smallest unit of training is a data pair rather than a single sample. Within a mini-batch of size *n*, the generalized contrastive loss compares every sample of one omics data type with every sample of the other, so the model is trained on *n*^2^ weighted pairs instead of *n* individual targets. The response supervises the relationships between sample pairs rather than each sample alone, which extracts more training signal from a limited number of subjects. We note that these pairs are formed from the same samples and are therefore not independent, so this makes the model better suited to modest sample sizes but does not remove the need for adequate data. MB-SupCon-cont performed well at the sizes studied here, but larger and more diverse datasets would provide a stronger test of generalizability, and the sample size needed for stable training deserves further study.

Our study also has limitations that point to future work. The Reverse PCA simulation, although validated against the real data, recovers only part of the feature variance and thus may be an optimistic model. It may also ignore sources of variation that real omics data can carry, such as batch effects. On the biological side, the associations we report are correlational, and a deeper mechanistic analysis would strengthen the biological interpretation. Linking microbes to metabolites within the shared embedding space, and uncovering associations beyond the direct ones, is a natural direction for future work.

In conclusion, MB-SupCon-cont, coupled with encoder-based neural networks, possesses a distinct advantage in the approximation of non-linear functions and the modeling of high-dimensional data. The versatility of the MB-SupCon-cont framework makes it applicable across a wide range of multi-omics scenarios, ultimately enhancing the efficacy of integrative prediction models.

## Data Availability

Publicly available datasets were analyzed in this study. This data can be found here: https://github.com/ya61sen/MB-SupCon-cont.
