# Supplementary material for: A generalized supervised contrastive learning framework for integrative multi-omics prediction models
Source: Front Microbiomes. 2026 Aug 13;5:1825540. doi: 10.3389/frmbi.2026.1825540 (PMC13518532; doi:10.3389/frmbi.2026.1825540)
Supplement: Supplementary file 1 [file DataSheet1.pdf]

# A Generalized Supervised Contrastive Learning Framework for Integrative Multi-omics Prediction Models


Sen Yang (sky5218@psu.edu)[1],

Shidan Wang (Shidan.Wang@UTSouthwestern.edu)[2],

Yiqing Wang (lucy@mail.smu.edu)[3],

Ruichen Rong (Ruichen.Rong@UTSouthwestern.edu)[2],

Bo Li (lib3@chop.edu)[4],

Andrew Y. Koh (Andrew.Koh@UTSouthwestern.edu) [5,6,7],

Guanghua Xiao (Guanghua.Xiao@UTSouthwestern.edu) [2,5],

Qiwei Li (Qiwei.Li@UTDallas.edu) [8],

Dajiang Liu (dajiang.liu@psu.edu)[1*],

Xiaowei Zhan (Xiaowei.Zhan@UTSouthwestern.edu) [2,5,9*]

[1]Department of Public Health Sciences, College of Medicine, Pennsylvania State University, Hershey, PA, 17033

[2]Quantitative Biomedical Research Center, Department of Population and Data Sciences, University of Texas Southwestern Medical Center, Dallas, TX, 75390

[3]Department of Statistics and Data Science, Southern Methodist University, Dallas, TX 75275

[4]Department of Pathology and Laboratory Medicine, University of Pennsylvania, Philadelphia, PA19104

[5]Harold C. Simmons Cancer Center, University of Texas Southwestern Medical Center, Dallas, Texas, 75390

[6]Department of Microbiology, University of Texas Southwestern Medical Center, Dallas, Texas, 75390

[7]Department of Pediatrics, University of Texas Southwestern Medical Center, Dallas, Texas, 75390

[8]Department of Mathematical Sciences, University of Texas at Dallas, Richardson, TX 75080

[9]Center for Genetics of Host Defense, University of Texas Southwestern Medical Center, Dallas, Texas, 75390

*To whom correspondence should be addressed

# Supplementary Text

## Supplementary Methods 1. Encoder architecture, training, and hyperparameter selection

### M1.1 Encoder architecture

Each omics data type has its own encoder, and the two encoders do not share weights. Each encoder is a multilayer perceptron. Its input layer has the same width as the number of features of that omics data type. The input layer is followed by a sequence of fully connected blocks. Every block applies a linear layer, one-dimensional batch normalization, a ReLU6 activation, and dropout. A final linear layer maps to the embedding of dimension $d_e$.

We used $d_e = 10$ for the two real datasets. For the simulation study we varied $d_e$ over $\{10, 20, 40\}$ and report $d_e = 20$ as the representative setting.

The hidden-layer (excluding the input and output layers) widths were set to the number of features and then reduced in steps toward the embedding dimension. The higher-dimensional omics data were given more layers, so that the reduction proceeds gradually. The hidden-layer widths of the four encoders were:

- T2D (Zhou et al. 2019) microbiome (96 features): 64, 32
- T2D metabolome (724 features): 256, 64
- HFD (Morton et al. 2019)microbiome (913 features): 256, 64, 32
- HFD metabolome (11978 features): 2048, 512, 128, 32

For the simulation study, the two encoders each used a single hidden layer, whose width was set halfway between the number of input features and the embedding dimension. Because the embedding dimension varied over $\{10, 20, 40\}$, this hidden width varied with it:

- Omics-1 (100 features): 55, 60, 70 for $d_e = \{10, 20, 40\}$
- Omics-2 (500 features): 255, 260, 270 for $d_e = \{10, 20, 40\}$

### M1.2 Hyperparameter selection

For each dataset, response, and weighting scheme, we selected the dropout rate, the weight decay, and the temperature by the validation error, averaged over the four prediction heads and six tuning seeds. Because one hyperparameter set trains both encoders and the two omics sit on very different RMSE scales, we normalized each omics' error by its own grid minimum and averaged the two. Therefore, both omics drove the choice rather than the microbiome alone. The grid search used only the training and validation data, and the testing data were never used for selection. The search grid had $4 \times 4 \times 5 = 80$ combinations:

- Dropout: {0.2, 0.4, 0.6, 0.8}
- Weight decay: {0.0025, 0.0126, 0.0635, 0.32}
- Temperature: {0.125, 0.25, 0.5, 1.0, 2.0}

The selected values are given in **Supplementary Table 2**. The three weighting schemes preferred different settings. In particular, the exponential and negative-log schemes tended to select smaller temperatures than the linear scheme. This is consistent with the more concentrated weights that these two schemes produce.

### M1.3 Sensitivity and stability of the selected hyperparameters

We examined how much the validation RMSE changed across the grid, and how stable the selected settings were across seeds. Within each study, response, and scheme, the validation RMSE varied in a moderate

range across the grid. For most cells, the best and the worst settings differed by a factor of roughly 1.2 to 1.6. Dropout and weight decay had a larger effect than temperature on the low-dimensional microbiome data, while temperature became more influential for the exponential and negative-log schemes.

Across the six tuning seeds, the best grid cell was not always identical, but the top settings were consistent. The ranking of grid cells by validation RMSE was positively correlated across seeds, with a mean pairwise Spearman correlation mostly between 0.5 and 0.9. The setting chosen on one seed usually fell in the top decile of the grid on the other seeds. The tuning is therefore stable and does not depend on a single lucky seed. **Supplementary Table 3** reports these values for every cell.

## Supplementary Methods 2. Baseline models

We compared MB-SupCon-cont against three groups of baselines. All baselines used the same 12 training-validation-testing splits, the same standardized omics data, and the same four regression heads as when the input is embeddings. The comparison is therefore made with identical prediction models on the same underlying data, and the methods differ only in the input fed to those models. We used the original features for the classical baselines and a lower-dimensional embedding for the others. For every method, we report the RMSE of the best head for each omics data type in **Table 1** and **Supplementary Table 1.**

### M2.1 Tuned classical baselines

The classical baselines were elastic net, support vector regression, random forest, and extreme gradient boosting (Chen and Guestrin 2016), fit on the original omics data. Each baseline was tuned by 5-fold cross-validation on the combined training and validation data, with the testing data held out. The cross-validation used `KFold` with 5 folds, shuffling, and a fixed random state. The model with the lowest cross-validation error was refit and then evaluated once on the testing data. The search grids were:

- Elastic net: `alpha` in {0.001, 0.01, 0.1, 1}, `l1_ratio` in {0.2, 0.5, 0.8}, max_iter = 10000
- Support vector regression (RBF kernel): `C` in {0.1, 1, 10}, `gamma` in {scale, auto}
- Random forest: `n_estimators` in {100, 300}, `max_depth` in {none, 10, 20}, `min_samples_leaf` in {1, 5}
- Extreme gradient boosting: `n_estimators` in {100, 300}, `max_depth` in {3, 6}, `learning_rate` in {0.05, 0.1}

We implemented these baselines with scikit-learn (Pedregosa et al. 2011), using its `ElasticNet`, `SVR`, and `RandomForestRegressor` classes, and the `xgboost` package for extreme gradient boosting through its `XGBRegressor` class. The cross-validated tuning used scikit-learn's `GridSearchCV`. The settings listed above are arguments of these classes, and every argument not listed was left at its package default.

### M2.2 Canonical correlation analysis

As a multi-view baseline, we fit canonical correlation analysis on the training data, using the same number of components as the embedding dimension. The canonical variates of each omics data type were passed to the same regression heads. We implemented canonical correlation analysis (HOTELLING 1936) with the `CCA` class of scikit-learn (`sklearn.cross_decomposition.CCA`), with `n_components` set to the embedding dimension and `max_iter` raised to 2000 for convergence. These settings are arguments of that class, and the remaining arguments were left at their defaults.

### M2.3 Autoencoder

To separate the contribution of the contrastive loss from that of the neural network, we trained two independent autoencoders, one per omics data type. Each uses the same encoder as MB-SupCon-cont,

followed by a decoder that mirrors it in reverse, and is trained to reconstruct its own omics data by minimizing the mean squared reconstruction error with no cross-omics coupling. The encoder outputs were used as representations and passed to the same regression heads. The autoencoder therefore shares the architecture and the capacity of MB-SupCon-cont and differs from it only in the training objective.

The autoencoder optimizes reconstruction rather than the response, so it was tuned on its own objective. We selected its dropout rate (0 or 0.2) and its weight decay (0, $10^{-5}$, or $10^{-4}$) by the lowest mean validation reconstruction loss over three seeds, separately for each study. Every other setting was kept identical to MB-SupCon-cont, including the embedding dimension of 10, stochastic gradient descent with a learning rate of 0.001 and a momentum of 0.9, gradient clipping, a batch size of 32, 1000 epochs, and the same 12 splits. The selected settings were a dropout rate of 0 with weight decay $10^{-5}$ for the T2D study, and a dropout rate of 0 with weight decay 0 for the HFD study. Because the two methods share an architecture, and each was tuned on the objective it optimizes, the difference between them is attributable to the contrastive objective rather than to the network.

## Supplementary Methods 3. The Reverse PCA algorithm

Reverse PCA reconstructs original omics data from a lower-dimensional representation. It assumes that for two sets of omics data there is a representation space in which the corresponding features of the two datasets are correlated. The components of the low-dimensional representation are treated as the highest-variance principal components. Reverse PCA multiplies the representation by principal component loadings, which reconstructs a reduced-rank approximation of the original data. The response is then generated from the same representation. The representation therefore carries the shared, response-related signal that MB-SupCon-cont is designed to recover. The detailed generative steps are given in **Algorithm 1**.

## Supplementary Methods 4. Additional simulation regimes and Reverse PCA validation

### M4.1 Additional simulation regimes

Reverse PCA builds the response from the same kind of low-dimensional representation that MB-SupCon-cont is designed to recover. To test whether the advantage depends on this design, we added three more realistic regimes. In all three, the data are generated exactly as in the baseline (**Algorithm 1**), with the embedding dimension fixed at 20 and the average feature correlation at 0.6, and only the response coefficients change. In the baseline, these coefficients, one for each of the 20 latent components of a layer, are drawn from $N(0, 25)$.

- Noise regime. Only a subset of the representation carries signal, and the remaining components are uninformative noise. We keep the first 5 of the 20 components in the response and set the coefficients of the other 15 to zero, while the full representation still generates the omics data.
- Single informative omics regime. Only one omics layer is informative. We ran two versions, one in which the Omics-1 layer carries the signal and one in which the Omics-2 layer carries it. In each version we set the entire coefficient vector of the uninformative layer to zero, which also removes the cross-omics interaction term.
- Sparse regime. The response coefficients are sparse, so only a few directions of the representation drive the response. We multiply each layer's coefficient vector by an independent mask that equals 1 with probability 0.2 and 0 otherwise, which leaves about 4 of the 20 coefficients nonzero.

As in the main simulation, the two simulated layers are named Omics-1 and Omics-2. The simulated data are generated from a latent representation and carry no biological identity.

**Algorithm 1** Simulation via Reverse PCA

Denote the dimension of original data as $D_x, D_y$, the dimension of embedding as $d_e$, the average correlation of features as $\mu_\rho$ and the standard deviation of correlation as $\sigma_\rho^2$.

**Step 1: Generate embeddings.**

1: **for** $k = 1, 2, ..., d_e$ **do**

2: Generate embeddings ${\mathbf{z}_\mathbf{x}}^{(k)}, {\mathbf{z}_\mathbf{y}}^{(k)}$ for omics data $\mathbf{x}$ and $\mathbf{y}$, respectively, s.t.,

$$({\mathbf{z}_\mathbf{x}}^{(k)}, {\mathbf{z}_\mathbf{y}}^{(k)}) \sim \text{MVN}(\boldsymbol{\mu}^{(k)}, \Sigma^{(k)}) \quad \text{with } \Sigma^{(k)} = \begin{pmatrix} 1 & \rho^{(k)} \\ \rho^{(k)} & 1 \end{pmatrix}$$

where the correlation of corresponding embedding features $\rho^{(k)} \sim N(\mu_\rho, \sigma_\rho^2)$ and $\Sigma^{(k)}$ needs to be positive semi-definite.

3: **end for**

4: **return** Embedding matrices

$$\mathbf{Z}_\mathbf{x} = [{\mathbf{z}_\mathbf{x}}^{(1)}, {\mathbf{z}_\mathbf{x}}^{(2)}, ..., {\mathbf{z}_\mathbf{x}}^{(d_e)}],\ \mathbf{Z}_\mathbf{y} = [{\mathbf{z}_\mathbf{y}}^{(1)}, {\mathbf{z}_\mathbf{y}}^{(2)}, ..., {\mathbf{z}_\mathbf{y}}^{(d_e)}].$$

**Step 2: Generate original data.**

1: Generate diagonal matrices with ordered explained variances for each principal component

$$\mathbf{V}_\mathbf{x} = \text{diag}(v_{(1)}, v_{(2)}, ..., v_{(D_x)}), \quad \mathbf{V}_\mathbf{y} = \text{diag}(v'_{(1)}, v'_{(2)}, ..., v'_{(D_y)}).$$

where the unordered explained variances before exaggeration are generated from an exponential distribution: $v_i^{1/c} \sim \text{Exp}(\lambda)$ for $i = 1, 2, ..., D_x$. with scale parameter $\lambda = 1$ and an exaggeration factor $c = 3$, and similarly ${v'_j}^{1/3} \sim \text{Exp}(1)$ for $j = 1, 2, ..., D_y$.

2: Generate orthogonal matrices $\mathbf{Q}_{\mathbf{x}(D_x \times D_x)}$ and $\mathbf{Q}_{\mathbf{y}(D_y \times D_y)}$ by applying QR decompositions to normally distributed square matrices.

3: Loading matrices are:

$$\mathbf{L}_\mathbf{x} = \mathbf{Q}_\mathbf{x}\sqrt{\mathbf{V}_\mathbf{x}}, \quad \mathbf{L}_\mathbf{y} = \mathbf{Q}_\mathbf{y}\sqrt{\mathbf{V}_\mathbf{y}}.$$

4: Retain only the first $d_e$ principal components and discard the remaining lower-variance principal components:

$${\mathbf{L}_\mathbf{x}}^{(\text{reduced})} = \mathbf{L}_\mathbf{x}[:, 1:d_m], {\mathbf{L}_\mathbf{y}}^{(\text{reduced})} = \mathbf{L}_\mathbf{y}[:, 1:d_m]$$

5: **return** Reduced-rank approximations of the standardized original omics data:

$$\mathbf{X} = \mathbf{Z}_\mathbf{x}[{\mathbf{L}_\mathbf{x}}^{(\text{reduced})}]^T, \quad \mathbf{Y} = \mathbf{Z}_\mathbf{y}[{\mathbf{L}_\mathbf{y}}^{(\text{reduced})}]^T.$$

**Step 3: Generate a response variable.**

1: Generate coefficient vectors $\boldsymbol{\beta}_{\boldsymbol{x}(d_e \times 1)}, \boldsymbol{\beta}_{\boldsymbol{y}(d_e \times 1)}$.

2: Suppose the response is quadratically associated with both omics data embeddings.

3: **return** The response variable

$$\begin{aligned}\mathbf{r} = \mathbf{Z}_\mathbf{x}\text{diag}(\boldsymbol{\beta}_{\boldsymbol{x}}) + \mathbf{Z}_\mathbf{y}\text{diag}(\boldsymbol{\beta}_{\boldsymbol{y}}) + \mathbf{Z}_\mathbf{x} \circ \mathbf{Z}_\mathbf{x}\text{diag}(\boldsymbol{\beta}_{\boldsymbol{x}}) + \mathbf{Z}_\mathbf{y} \circ \mathbf{Z}_\mathbf{y}\text{diag}(\boldsymbol{\beta}_{\boldsymbol{y}}) + \\ \mathbf{Z}_\mathbf{x} \circ \mathbf{Z}_\mathbf{y}\text{diag}(\boldsymbol{\beta}_{\boldsymbol{x}})\text{diag}(\boldsymbol{\beta}_{\boldsymbol{y}}) + \epsilon.\end{aligned}$$

where $\circ$ denotes the Hadamard product and the random noise $\epsilon \sim N(0, 1)$.

**Step 4: MB-SupCon-cont.**

1: Input $(\mathbf{X}, \mathbf{Y})$ to MB-SupCon-cont and make predictions on $\mathbf{r}$.

**Supplementary Table 4** reports the best RMSE over the four regression heads, for the original data and for the embeddings from the three weighting schemes in each regime. **Supplementary Figure 6** shows the corresponding distributions over the 12 replicates.

In the baseline, single informative omics, and sparse regimes, the embeddings gave lower prediction error than the original data. The reduction was largest for the informative omics layer. When only the Omics-1 layer carried signal, the Omics-1 embedding RMSE dropped from about 9.13 to 4.38. When only the Omics-2 layer carried signal, the Omics-2 embedding RMSE dropped from about 7.41 to 3.55. In both cases the uninformative layer showed no gain, as expected.

In the noise regime, most of the representation is uninformative, and the advantage of the embeddings shrank to roughly a tie at the best head. This is the expected behavior, because there is little shared structure left for a contrastive model to exploit.

### M4.2 Reverse PCA validation on the real data

Reverse PCA assumes that a real omics dataset can be summarized by its leading principal components. The way to test that assumption is to perform the summary and then measure what is lost. For each omics data type in each real dataset, we standardized every feature, kept the top $k$ principal components, and reconstructed the data from them. We then measured, for every feature, the fraction of its variance that the reconstruction recovers, which is the reconstruction $R^2$ of that feature. We used $k = 10$, 20, and 40, which are the embedding dimensions used in the simulation.

At an embedding dimension of 20, the reconstruction recovered about 79% of the feature variance for the T2D microbiome, 49% for the T2D metabolome, 34% for the HFD microbiome, and 46% for the HFD metabolome (**Supplementary Table 5**). Recovery improved with $k$, as expected. **Supplementary Figure 7** shows the full distribution of the per-feature reconstruction $R^2$ at each $k$, which makes visible both how much of each dataset is recovered and how much is lost.

The leading components therefore capture a substantial part of the low-dimensional structure of the real data, but they do not capture all of it. The recovery is uneven across the four datasets, and it is weakest for the HFD study, where the number of features greatly exceeds the number of samples. Reverse PCA is thus a reasonable but optimistic generative model of real omics data, and the simulated data are cleaner and lower dimensional than the data they imitate. The features with the poorest reconstruction were mainly low-abundance taxa and metabolites, which principal component analysis captures least well.

## Supplementary Results 1. Prediction error and significance testing

### R1.1 Per-head prediction results

**Supplementary Tables 14 to 23** give the full results, listing every response, omics data type, and prediction head separately, for the original data and for the embeddings from all three weighting schemes.

### R1.2 Full significance testing

**Supplementary Table 6** gives every pairwise comparison. Each row compares MB-SupCon-cont, at the best weighting scheme and head for that cell, with one comparator at its own best head. We report the mean difference in RMSE, a 95% paired bootstrap confidence interval for that difference, the paired Wilcoxon signed-rank p-value over the 12 splits, and the Benjamini-Hochberg adjusted p-value (Benjamini and Hochberg 1995).

## Supplementary Results 2. Variance explained by principal component analysis

The principal component analysis scatter plots in the main text (**Figure 4**) and the supplementary (**Supplementary Figure 4 and 5**) show only the first two components. Their value as a summary depends on how much of the variance those two components carry. **Supplementary Table 7** reports that share, for the original data and for the MB-SupCon-cont embeddings. These are the explained-variance ratios of the training-set principal component analysis, the same one used to draw the scatter plots, which is fitted on the training split and then used to project the samples shown.

For the original omics data, the first two components explain only a small share of the variance. This is expected, because the data are high dimensional and their variance is spread across many components. For the MB-SupCon-cont embeddings, the first two components explain a much larger share, so the two-dimensional scatter plots are a faithful view of the embedding. Among the three schemes, the linear scheme concentrates the variance most strongly. This is consistent with its low effective dimensionality, reported in **Supplementary Results 4**.

For each omics data type and response, the scatter plots show that the embedding from the weighting method that performed best for that cell. The first two components still explain a large share of the variance of that embedding. For the T2D metabolome, they explain about 0.83 of the variances for Age with linear weights, 0.59 for BMI, and 0.64 for SSPG with negative-log weights, compared with about 0.14 to 0.16 in the original data.

## Supplementary Results 3. Weighting-scheme selection and noise robustness

This section gives the full analysis behind the main-text subsection on choosing a weighting scheme.

### R3.1 Weighting scheme and response range

We first asked whether the best scheme could be read off a simple property of the response, its range, which would remove the need for a data-driven rule. It cannot: across the ten real cells the test RMSE of all three schemes rises with the response range in almost the same way, with a Spearman correlation between the response range and the linear, exponential, and negative-log RMSE of 0.84, 0.84, and 0.86 respectively (each $p < 0.01$). The range therefore shifts the three schemes together and does not separate them, so the choice of scheme is not a function of the response scale and has to be made from the data (**Supplementary Table 8**).

### R3.2 The validation-based selection rule

For each dataset, response, and split, we took each scheme at its best prediction head, which is the head with the lowest mean test RMSE reported in **Table 1**. We then selected the scheme with the lowest validation RMSE. We compared this rule against the oracle, which is the scheme with the lowest test RMSE on that same split. Across the ten real-data cells, the scheme the rule selected most often matched the best-performing scheme in nine. The rule also stayed close to the oracle, with a mean regret (the test RMSE of the scheme the rule selects minus the test RMSE of the scheme that performs best on the test data) of about 0.49 RMSE units across these cells, and 0.33 when the simulation regimes are included. The one exception was HFD Weight in the metabolome, where the rule most often chose the exponential scheme while the linear scheme was slightly better, by about 0.2 RMSE. **Supplementary Table 9** gives the per-cell values for the real datasets.

### R3.3 Response-noise robustness

We then studied how each scheme degrades when the response contains measurement error. We added Gaussian noise to the training and validation responses, in steps up to a level equal to one standard deviation of the response, while keeping the test response clean. At a noise fraction $f$, each response value $y$ became $y + f \cdot \mathrm{std}(y) \cdot z$, where $z$ is a standard normal draw. The five fractions were $f = \{0, 0.1, 0.25, 0.5, 1.0\}$. **Supplementary Table 10** reports the clean and the fully noised RMSE for each scheme, together with the absolute and the relative increase.

For three of the five responses, namely T2D Age, T2D BMI, and HFD Age, the ranking of the schemes was stable as the noise increased. For the other two responses the ranking flipped under heavy noise. For T2D SSPG, which has by far the widest response range, the exponential scheme became the most robust, with an RMSE of 46.15 at full noise against 54.55 for linear weights. For HFD Weight, the negative-log scheme became the most robust, with an RMSE of 8.21 against 10.27 for linear weights.

The explanation lies in how the schemes treat distant pairs. The exponential and negative-log schemes down-weight distant pairs more strongly, so they rely on the closest pairs, whose ordering noise is least likely to disturb. The validation-based rule picks these schemes automatically in the noisy cells, without being told that the response is noisy.

## Supplementary Results 4. Embedding geometry and effective dimensionality

To test whether the embeddings are organized by the response, we measured the Mantel correlation (Mantel 1967) between the pairwise embedding distances and the pairwise response distances, with 999 permutations. The MB-SupCon-cont embeddings tracked the response far more closely than principal component analysis or the autoencoder did, which shows that this structure comes from the supervision and not from the omics data alone.

To test whether the three weighting schemes build the same representation or different ones, we measured the effective dimensionality of each embedding as the participation ratio (PR). It is defined as $\mathrm{PR} = \frac{(\sum_i \lambda_i)^2}{\sum_i \lambda_i^2}$ , where the $\lambda_i$ are the eigenvalues of the embedding covariance (the squared singular values), which ranges from 1 when the embedding collapses onto a single direction to $d_e$ when its variance is spread evenly across all directions. The schemes spread over clearly different numbers of dimensions, from about 1 to 2 for the concentrated linear scheme up to about 7 for the other two, all well below the roughly 6.8 to 8.4 of the original data, so they are genuinely different geometries rather than reweighted copies of one. **Supplementary Table 11** reports both measures for every study, omics data type, and response.

## Supplementary Results 5. Full ranked biological associations

**Supplementary Table 12** extends main-text Table 2 to the full top 20 along each response axis and to both omics data types, so the metabolites and the remaining microbes can be read next to the leading microbes. It shows that the embedding-axis ranking and the direct ranking agree well down the list, and it gives the split recurrence that measures how stable that agreement is.

**Supplementary Table 13** is the evidence that this recovery comes from the supervision and not from the omics data. For every cell it compares MB-SupCon-cont with a shuffled-response encoder, the autoencoder, and principal component analysis, on how well the response axis tracks the held-out response and how many of the directly associated top 20 features it recovers. MB-SupCon-cont led all three in every cell, while the shuffled-response encoder, which differs only in that its supervision was destroyed, stayed near the floor. This is the per-cell detail behind main-text Figure 7.

# Supplementary Figures

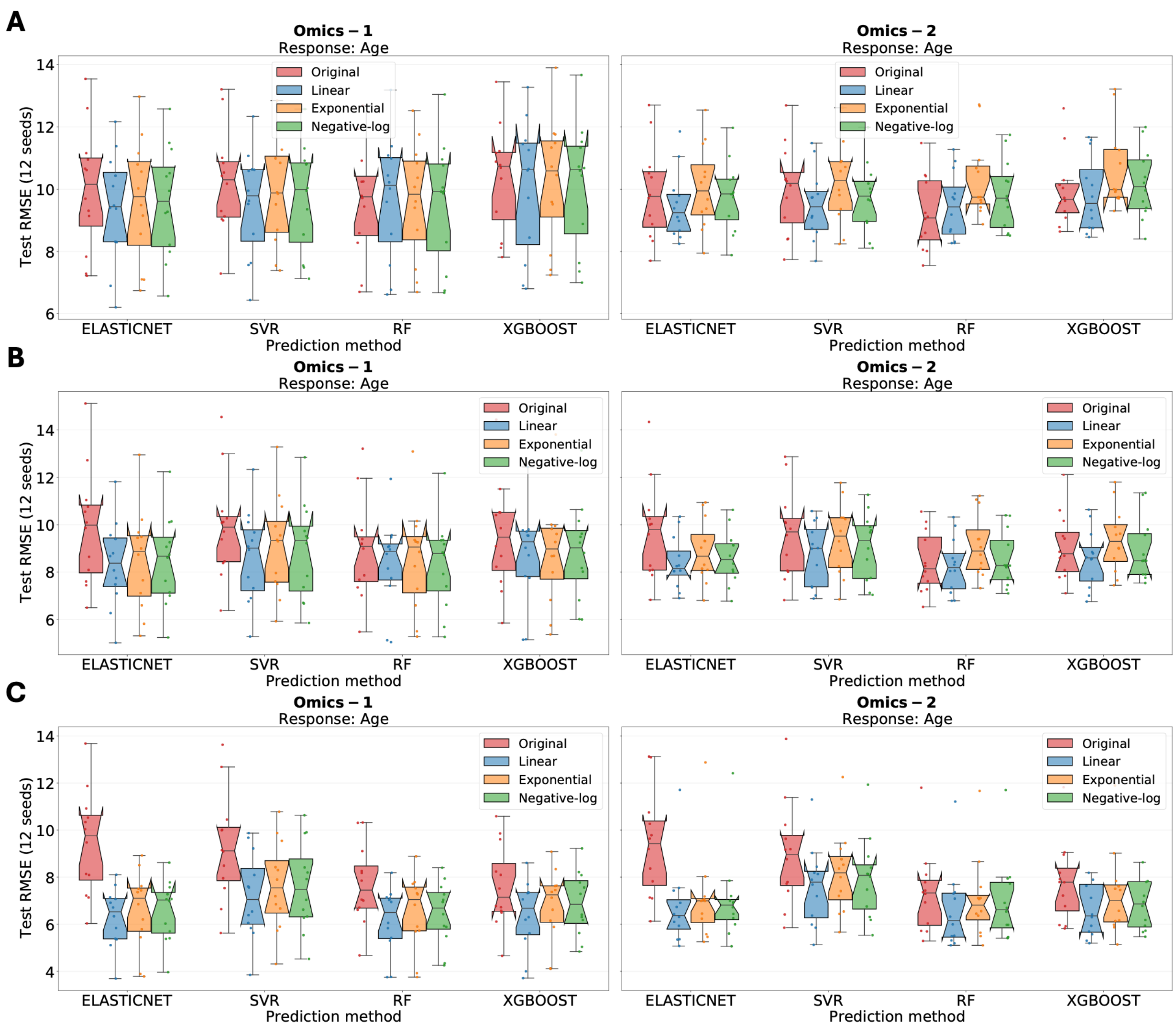


**Supplementary Figure 1.** Simulation study, embedding dimension 10. Boxplots of prediction root-mean-square errors (RMSEs) on testing data (15%) of the simulation study from 12 random replicates when the dimension of embeddings is 10. Panel A: $\mu_\rho = 0.4$; Panel B: $\mu_\rho = 0.6$; Panel C: $\mu_\rho = 0.8$. Within each panel the two simulated omics data types, Omics-1 and Omics-2, are shown side by side. The red boxes are the prediction on original data and the other boxes are the prediction on embeddings. Colors: red, original data; blue, linear; orange, exponential; green, negative-log. Points are the 12 per-seed RMSEs.

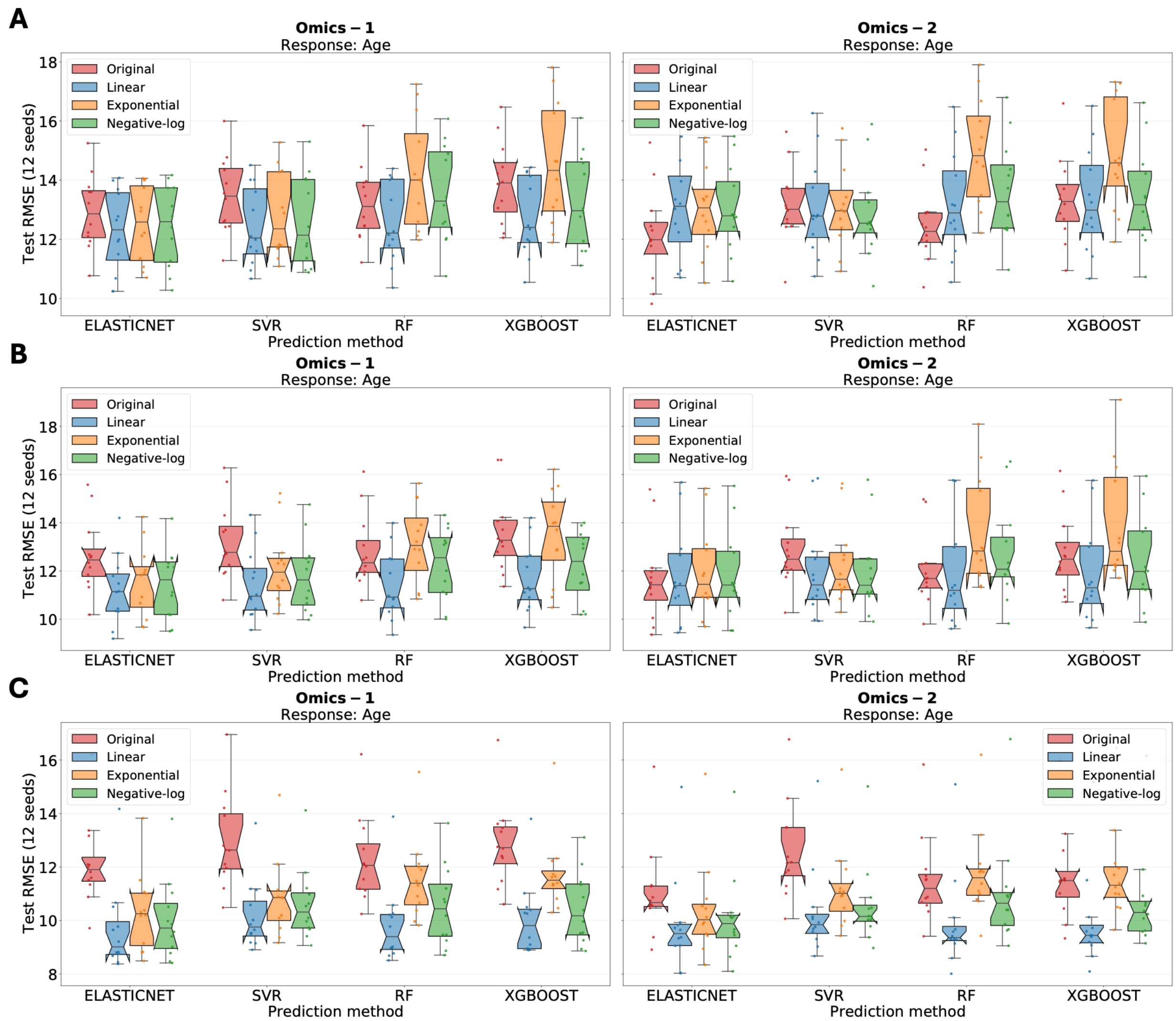


**Supplementary Figure 2.** Simulation study, embedding dimension 40. Boxplots of prediction RMSEs on testing data (15%) of the simulation study from 12 random replicates when the dimension of embeddings is 40. Panel A: $\mu_\rho = 0.4$; Panel B: $\mu_\rho = 0.6$; Panel C: $\mu_\rho = 0.8$. Within each panel the two simulated omics data types, Omics-1 and Omics-2, are shown side by side. The red boxes are the prediction on original data and the other boxes are the prediction on embeddings. Colors: red, original data; blue, linear; orange, exponential; green, negative-log. Points are the 12 per-seed RMSEs.

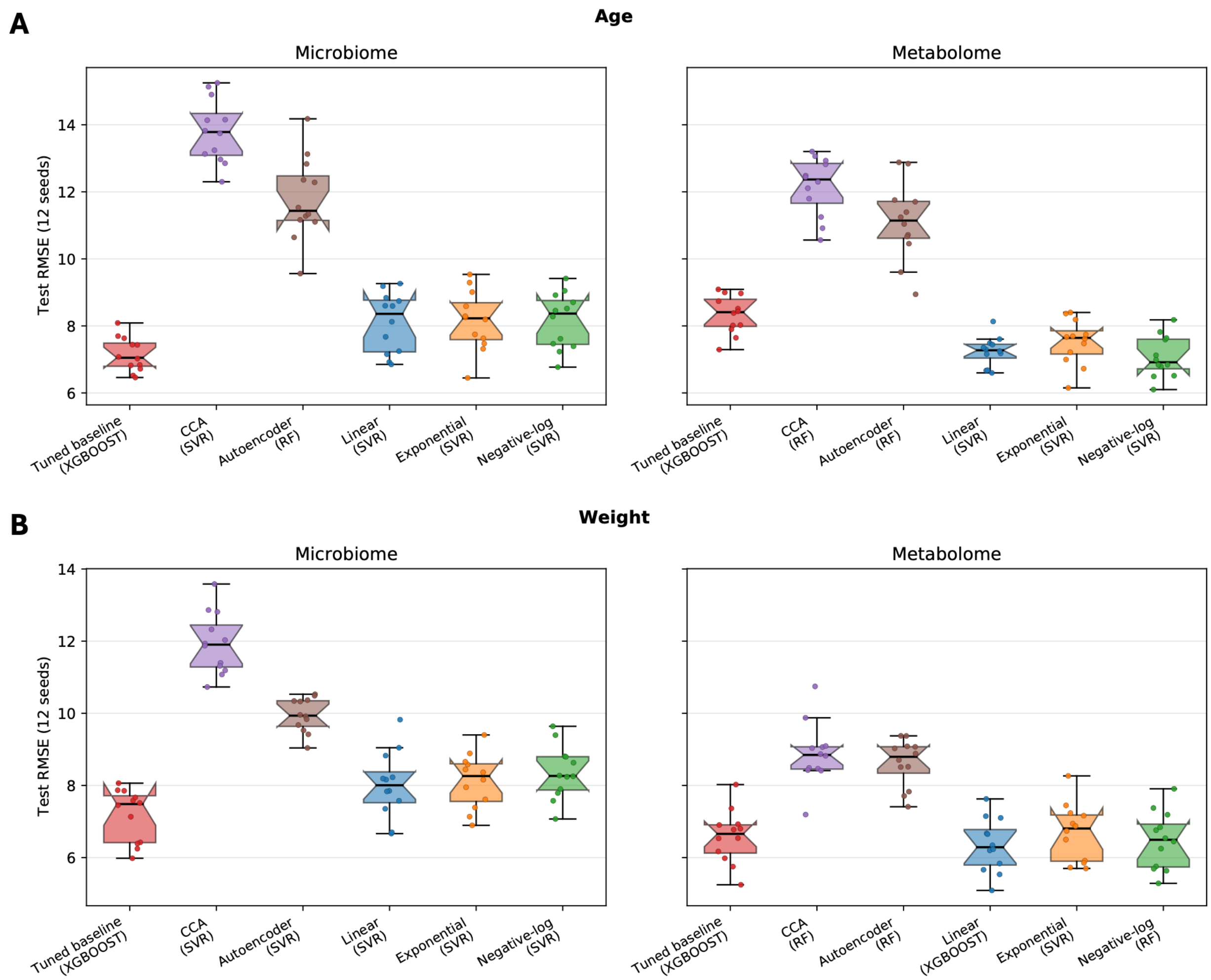


**Supplementary Figure 3.** Prediction error in the HFD study. Boxplots of prediction RMSEs on testing data from 12 random training-validation-testing splits, by using different methods for the two continuous responses (HFD study). Panel A: Age; Panel B: Weight. Each method is shown at its best-performing prediction head, given in parentheses below the box. Within each panel the two omics data types are side by side on a common y axis, microbiome left and metabolome right. Boxes are notched at the median and the points are the 12 per-seed RMSEs. Colors: red, tuned baseline; purple, canonical correlation analysis; brown, autoencoder; blue, linear; orange, exponential; green, negative-log.

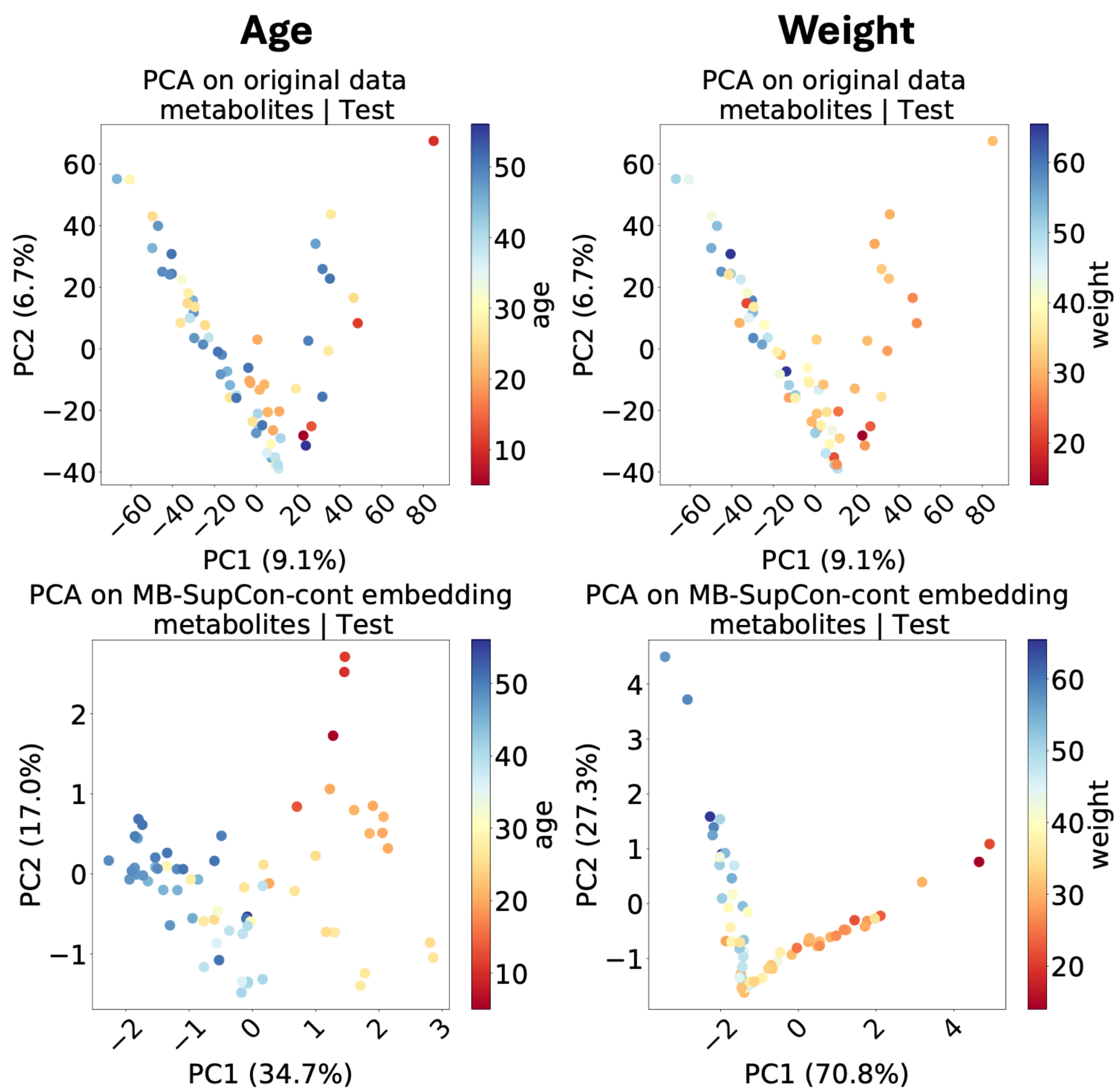


**Supplementary Figure 4.** HFD metabolome, original data and embeddings. PCA scatter plots (PC2 vs. PC1) on original metabolome data vs. MB-SupCon-cont metabolome embeddings (HFD study) for responses Age and Weight, based on testing data. First row: original data; second row: embeddings, each shown at the weighting method that performed best for that omics and response, which for the metabolome is negative-log for Age and linear for Weight.

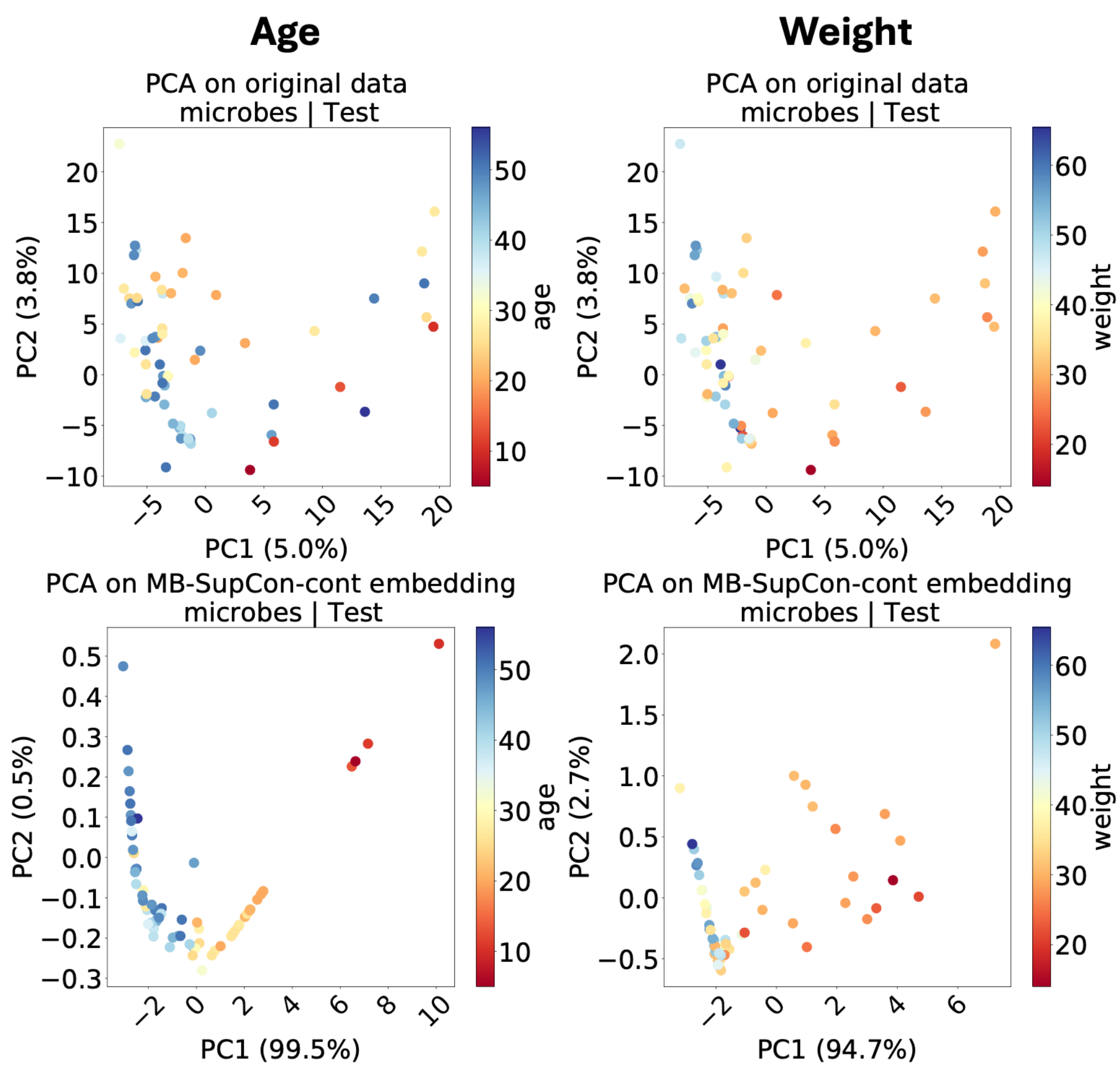


**Supplementary Figure 5.** HFD microbiome, original data and embeddings. PCA scatter plots (PC2 vs. PC1) on original microbiome data vs. MB-SupCon-cont microbiome embeddings (HFD study) for responses Age and Weight, based on testing data. First row: original data; second row: embeddings, each shown at the weighting method that performed best for that omics and response, which for the microbiome is linear for both Age and Weight.

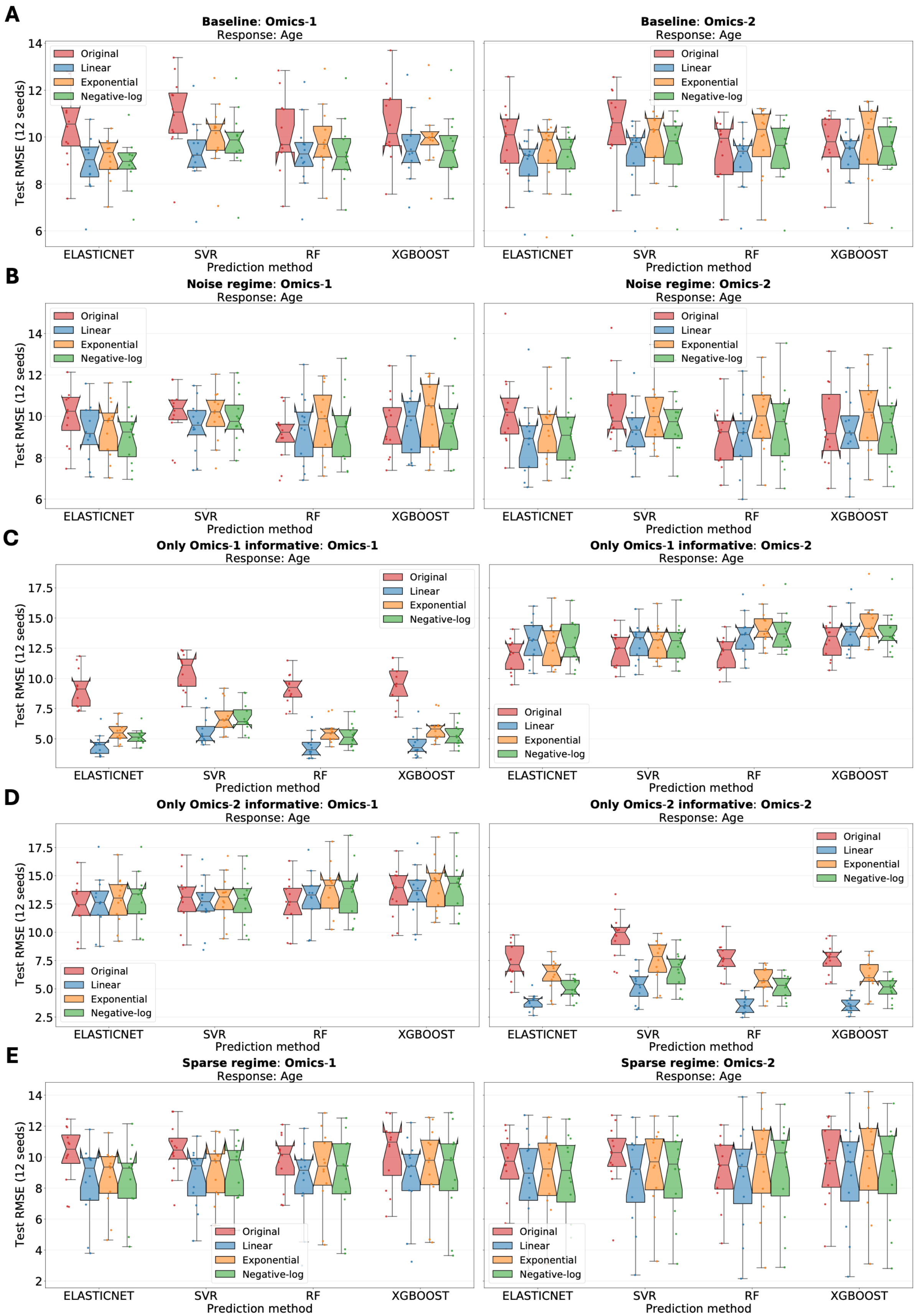
A
Baseline: Omics-1
Response: Age
Baseline: Omics-2
Response: Age
B
Noise regime: Omics-1
Response: Age
Noise regime: Omics-2
Response: Age
C
Only Omics-1 informative: Omics-1
Response: Age
Only Omics-1 informative: Omics-2
Response: Age
D
Only Omics-2 informative: Omics-1
Response: Age
Only Omics-2 informative: Omics-2
Response: Age
E
Sparse regime: Omics-1
Response: Age
Sparse regime: Omics-2
Response: Age
Original
Linear
Exponential
Negative-log
Test RMSE (12 seeds)
ELASTICNET
SVR
RF
XGBOOST
Prediction method

**Supplementary Figure 6.** Simulation under the three additional regimes. Boxplots of prediction RMSEs on testing data for the three additional simulation regimes, comparing the original data with the embeddings from the three weighting methods. Panel A: baseline reference. Panel B: noise regime. Panel C: single informative omics, Omics-1 informative. Panel D: single informative omics, Omics-2 informative. Panel E: sparse regime. Within each panel the two simulated omics data types, Omics-1 and Omics-2, are shown side by side.

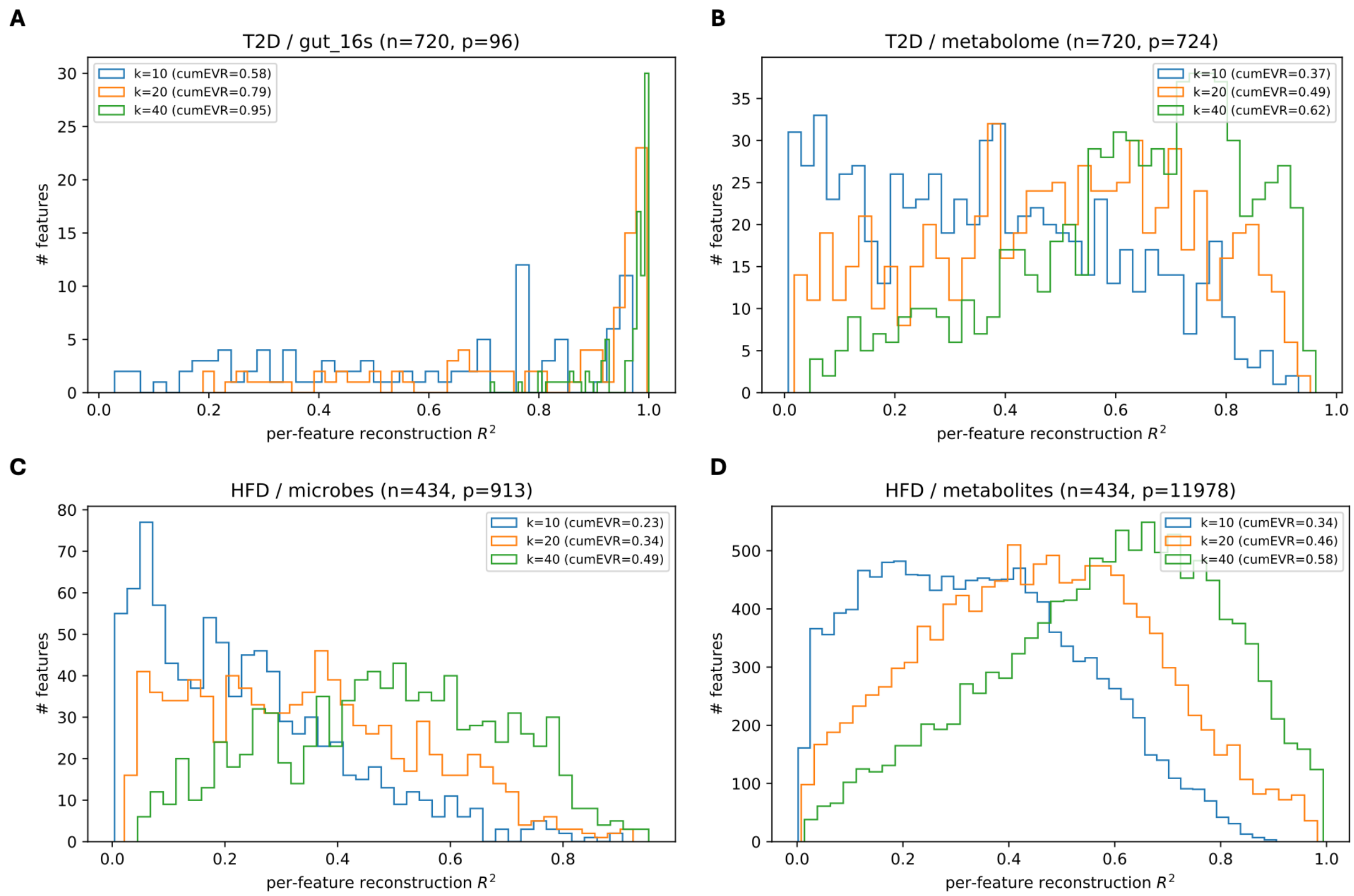


**Supplementary Figure 7.** Reverse PCA reconstruction quality on the real data. Histograms of per-feature reconstruction $R^2$ for the real omics data. Panel A: T2D microbiome. Panel B: T2D metabolome. Panel C: HFD microbiome. Panel D: HFD metabolome. Each panel shows the distribution at $k$ = 10, 20, and 40, with the cumulative explained variance ratio for each $k$ in the legend.

# Supplementary Tables

**Supplementary Table 1.** Prediction error in the HFD study. Average RMSEs on testing data (HFD study) for predicting Age and Weight by different prediction methods, based on original omics data and MB-SupCon-cont embeddings using the three weighting methods (italic). The table compares the best tuned classical baseline (with the prediction head that achieved it), canonical correlation analysis (CCA), the autoencoder, and MB-SupCon-cont with the three weighting methods. For predictions based on embeddings, each weighting method is shown with its best-performing prediction head, given in parentheses. The best RMSE for each prediction task is in bold. Values are means over 12 random splits.

| Response | Tuned baseline | CCA | Autoencoder | *Linear* | *Exponential* | *Negative-log* |
|---|---|---|---|---|---|---|
| **Microbiome** | | | | | | |
| Age | 7.1453 (XGBoost) | 13.8022 (SVR) | 11.7820 (RF) | 8.0994 (SVR) | 8.1475 (SVR) | 8.1510 (SVR) |
| Weight | 7.1812 (XGBoost) | 11.9269 (SVR) | 9.9513 (SVR) | 8.0214 (SVR) | 8.1225 (SVR) | 8.3642 (SVR) |
| **Metabolome** | | | | | | |
| Age | 8.3330 (XGBoost) | 12.1549 (RF) | 11.1029 (RF) | 7.2369 (SVR) | 7.5197 (SVR) | **7.0759 (SVR)** |
| Weight | 6.5848 (XGBoost) | 8.8735 (RF) | 8.6241 (RF) | **6.3413 (XGBoost)** | 6.6964 (SVR) | 6.4727 (RF) |

**Supplementary Table 2.** Selected hyperparameters. Selected hyperparameters (dropout, weight decay, temperature) for each study, response, and weighting scheme, chosen by the lowest validation RMSE.

| **Study** | **Weighting scheme** | **Response** | **Dropout** | **Weight decay** | **Temperature** |
|---|---|---|---|---|---|
| T2D | linear | Age | 0.2 | 0.0126 | 1.0 |
| T2D | linear | BMI | 0.2 | 0.0126 | 1.0 |
| T2D | linear | SSPG | 0.4 | 0.0635 | 1.0 |
| T2D | exponential | Age | 0.2 | 0.0126 | 0.125 |
| T2D | exponential | BMI | 0.2 | 0.0126 | 0.5 |
| T2D | exponential | SSPG | 0.2 | 0.0126 | 0.125 |
| T2D | negative-log | Age | 0.2 | 0.0635 | 0.125 |
| T2D | negative-log | BMI | 0.2 | 0.0635 | 0.25 |
| T2D | negative-log | SSPG | 0.4 | 0.0635 | 0.5 |
| HFD | linear | Age | 0.4 | 0.0635 | 0.125 |
| HFD | linear | Weight | 0.6 | 0.0126 | 1.0 |
| HFD | exponential | Age | 0.4 | 0.0635 | 0.125 |
| HFD | exponential | Weight | 0.6 | 0.0635 | 0.125 |
| HFD | negative-log | Age | 0.2 | 0.0635 | 0.25 |
| HFD | negative-log | Weight | 0.6 | 0.0025 | 0.5 |

**Supplementary Table 3.** Stability of the selected hyperparameters across seeds. Stability of the selected hyperparameters across the 6 tuning seeds, for every study, weighting scheme, omics data type, response, and prediction head. For each cell the table gives the modal best setting (dropout, weight decay, temperature) and the number of seeds that chose it (model agreement), the mean pairwise Spearman correlation between the grid rankings of two seeds, and the top-decile hit rate, which is how often the setting chosen on one seed fell in the best ten percent of the grid on the other seeds.

| Study | Weighting scheme | Omics | Response | Head | Modal best setting | Modal agreement | Mean Spearman | Top-decile rate |
|---|---|---|---|---|---|---|---|---|
| T2D | linear | Microbiome | Age | ElasticNet | 0.2, 0.0635, 0.5 | 2/6 | 0.72 | 0.50 |
| T2D | linear | Microbiome | Age | SVR | 0.2, 0.0126, 0.125 | 2/6 | 0.83 | 0.67 |
| T2D | linear | Microbiome | Age | RF | 0.2, 0.0126, 0.125 | 2/6 | 0.72 | 0.67 |
| T2D | linear | Microbiome | Age | XGBoost | 0.2, 0.0126, 0.25 | 1/6 | 0.78 | 0.67 |
| T2D | linear | Microbiome | BMI | ElasticNet | 0.2, 0.0635, 0.125 | 2/6 | 0.84 | 0.67 |
| T2D | linear | Microbiome | BMI | SVR | 0.2, 0.0025, 0.5 | 1/6 | 0.79 | 0.67 |
| T2D | linear | Microbiome | BMI | RF | 0.2, 0.0126, 0.25 | 1/6 | 0.79 | 0.67 |
| T2D | linear | Microbiome | BMI | XGBoost | 0.2, 0.0126, 0.5 | 2/6 | 0.80 | 0.67 |
| T2D | linear | Microbiome | SSPG | ElasticNet | 0.2, 0.0635, 0.125 | 2/6 | 0.60 | 0.83 |
| T2D | linear | Microbiome | SSPG | SVR | 0.2, 0.0635, 2 | 2/6 | 0.70 | 0.67 |
| T2D | linear | Microbiome | SSPG | RF | 0.2, 0.0126, 0.5 | 1/6 | 0.76 | 0.33 |
| T2D | linear | Microbiome | SSPG | XGBoost | 0.2, 0.0126, 0.25 | 1/6 | 0.79 | 0.67 |
| T2D | linear | Metabolome | Age | ElasticNet | 0.4, 0.0635, 0.125 | 3/6 | 0.93 | 0.83 |
| T2D | linear | Metabolome | Age | SVR | 0.2, 0.0126, 2 | 3/6 | 0.87 | 0.67 |
| T2D | linear | Metabolome | Age | RF | 0.2, 0.0126, 2 | 2/6 | 0.86 | 0.67 |
| T2D | linear | Metabolome | Age | XGBoost | 0.2, 0.0126, 2 | 3/6 | 0.85 | 0.67 |
| T2D | linear | Metabolome | BMI | ElasticNet | 0.2, 0.0635, 0.125 | 2/6 | 0.89 | 1.00 |
| T2D | linear | Metabolome | BMI | SVR | 0.2, 0.0126, 0.5 | 2/6 | 0.88 | 1.00 |
| T2D | linear | Metabolome | BMI | RF | 0.2, 0.0126, 1 | 1/6 | 0.86 | 0.83 |
| T2D | linear | Metabolome | BMI | XGBoost | 0.2, 0.0126, 0.25 | 1/6 | 0.88 | 0.83 |
| T2D | linear | Metabolome | SSPG | ElasticNet | 0.4, 0.0635, 2 | 3/6 | 0.81 | 0.83 |
| T2D | linear | Metabolome | SSPG | SVR | 0.2, 0.0635, 2 | 1/6 | 0.84 | 0.83 |
| T2D | linear | Metabolome | SSPG | RF | 0.2, 0.0126, 1 | 1/6 | 0.79 | 0.50 |
| T2D | linear | Metabolome | SSPG | XGBoost | 0.6, 0.0126, 2 | 2/6 | 0.78 | 0.67 |
| T2D | exponential | Microbiome | Age | ElasticNet | 0.2, 0.0126, 0.5 | 2/6 | 0.54 | 0.83 |
| T2D | exponential | Microbiome | Age | SVR | 0.2, 0.0126, 0.125 | 1/6 | 0.61 | 0.67 |
| T2D | exponential | Microbiome | Age | RF | 0.2, 0.0126, 0.5 | 2/6 | 0.61 | 0.83 |
| T2D | exponential | Microbiome | Age | XGBoost | 0.2, 0.0025, 0.125 | 2/6 | 0.60 | 0.50 |
| T2D | exponential | Microbiome | BMI | ElasticNet | 0.2, 0.0126, 0.25 | 1/6 | 0.52 | 0.50 |
| T2D | exponential | Microbiome | BMI | SVR | 0.2, 0.0126, 2 | 2/6 | 0.59 | 0.67 |
| T2D | exponential | Microbiome | BMI | RF | 0.6, 0.0025, 0.125 | 2/6 | 0.51 | 0.83 |
| T2D | exponential | Microbiome | BMI | XGBoost | 0.2, 0.0126, 0.125 | 2/6 | 0.65 | 0.83 |
| T2D | exponential | Microbiome | SSPG | ElasticNet | 0.2, 0.0635, 0.125 | 2/6 | 0.66 | 0.83 |
| T2D | exponential | Microbiome | SSPG | SVR | 0.4, 0.32, 0.125 | 2/6 | 0.44 | 0.50 |
| T2D | exponential | Microbiome | SSPG | RF | 0.2, 0.0025, 0.125 | 1/6 | 0.67 | 1.00 |

| Study | Weighting scheme | Omics | Response | Head | Modal best setting | Modal agreement | Mean Spearman | Top-decile rate |
|---|---|---|---|---|---|---|---|---|
| T2D | exponential | Microbiome | SSPG | XGBoost | 0.2, 0.0025, 0.125 | 1/6 | 0.65 | 0.67 |
| T2D | exponential | Metabolome | Age | ElasticNet | 0.2, 0.0635, 0.125 | 3/6 | 0.79 | 1.00 |
| T2D | exponential | Metabolome | Age | SVR | 0.2, 0.0126, 0.125 | 4/6 | 0.77 | 1.00 |
| T2D | exponential | Metabolome | Age | RF | 0.2, 0.0126, 0.25 | 3/6 | 0.83 | 0.83 |
| T2D | exponential | Metabolome | Age | XGBoost | 0.2, 0.0126, 0.25 | 3/6 | 0.76 | 0.83 |
| T2D | exponential | Metabolome | BMI | ElasticNet | 0.2, 0.32, 0.125 | 2/6 | 0.61 | 0.83 |
| T2D | exponential | Metabolome | BMI | SVR | 0.2, 0.0635, 0.125 | 2/6 | 0.82 | 0.67 |
| T2D | exponential | Metabolome | BMI | RF | 0.2, 0.0126, 0.125 | 1/6 | 0.50 | 0.33 |
| T2D | exponential | Metabolome | BMI | XGBoost | 0.2, 0.0025, 0.25 | 1/6 | 0.61 | 0.50 |
| T2D | exponential | Metabolome | SSPG | ElasticNet | 0.2, 0.0635, 0.25 | 2/6 | 0.68 | 0.83 |
| T2D | exponential | Metabolome | SSPG | SVR | 0.2, 0.0126, 2 | 2/6 | 0.39 | 0.50 |
| T2D | exponential | Metabolome | SSPG | RF | 0.2, 0.0025, 0.125 | 3/6 | 0.78 | 1.00 |
| T2D | exponential | Metabolome | SSPG | XGBoost | 0.2, 0.0025, 0.125 | 2/6 | 0.78 | 0.83 |
| T2D | negative-log | Microbiome | Age | ElasticNet | 0.2, 0.0126, 0.25 | 2/6 | 0.60 | 1.00 |
| T2D | negative-log | Microbiome | Age | SVR | 0.2, 0.0126, 0.25 | 1/6 | 0.66 | 0.67 |
| T2D | negative-log | Microbiome | Age | RF | 0.2, 0.0635, 0.25 | 2/6 | 0.62 | 0.67 |
| T2D | negative-log | Microbiome | Age | XGBoost | 0.2, 0.0126, 0.125 | 1/6 | 0.73 | 0.67 |
| T2D | negative-log | Microbiome | BMI | ElasticNet | 0.2, 0.0126, 0.25 | 1/6 | 0.60 | 0.83 |
| T2D | negative-log | Microbiome | BMI | SVR | 0.2, 0.0126, 0.125 | 1/6 | 0.56 | 0.67 |
| T2D | negative-log | Microbiome | BMI | RF | 0.2, 0.0025, 2 | 1/6 | 0.54 | 0.33 |
| T2D | negative-log | Microbiome | BMI | XGBoost | 0.2, 0.0025, 0.125 | 1/6 | 0.67 | 0.83 |
| T2D | negative-log | Microbiome | SSPG | ElasticNet | 0.2, 0.0635, 1 | 1/6 | 0.47 | 0.33 |
| T2D | negative-log | Microbiome | SSPG | SVR | 0.2, 0.0635, 1 | 2/6 | 0.47 | 0.67 |
| T2D | negative-log | Microbiome | SSPG | RF | 0.2, 0.0025, 2 | 1/6 | 0.60 | 0.33 |
| T2D | negative-log | Microbiome | SSPG | XGBoost | 0.2, 0.0025, 1 | 1/6 | 0.68 | 0.67 |
| T2D | negative-log | Metabolome | Age | ElasticNet | 0.2, 0.0635, 0.125 | 3/6 | 0.93 | 1.00 |
| T2D | negative-log | Metabolome | Age | SVR | 0.2, 0.0126, 0.5 | 3/6 | 0.92 | 1.00 |
| T2D | negative-log | Metabolome | Age | RF | 0.2, 0.0126, 1 | 1/6 | 0.81 | 0.50 |
| T2D | negative-log | Metabolome | Age | XGBoost | 0.2, 0.0126, 1 | 1/6 | 0.84 | 0.67 |
| T2D | negative-log | Metabolome | BMI | ElasticNet | 0.6, 0.0635, 0.125 | 2/6 | 0.74 | 0.83 |
| T2D | negative-log | Metabolome | BMI | SVR | 0.2, 0.0635, 0.25 | 2/6 | 0.79 | 1.00 |
| T2D | negative-log | Metabolome | BMI | RF | 0.2, 0.0635, 0.5 | 2/6 | 0.54 | 0.33 |
| T2D | negative-log | Metabolome | BMI | XGBoost | 0.2, 0.0635, 0.25 | 2/6 | 0.74 | 0.67 |
| T2D | negative-log | Metabolome | SSPG | ElasticNet | 0.2, 0.0635, 0.125 | 1/6 | 0.67 | 0.67 |
| T2D | negative-log | Metabolome | SSPG | SVR | 0.4, 0.0635, 1 | 2/6 | 0.84 | 1.00 |
| T2D | negative-log | Metabolome | SSPG | RF | 0.2, 0.0126, 0.5 | 1/6 | 0.62 | 0.50 |
| T2D | negative-log | Metabolome | SSPG | XGBoost | 0.2, 0.0126, 0.5 | 1/6 | 0.74 | 0.67 |
| HFD | linear | Metabolome | Age | ElasticNet | 0.2, 0.0635, 0.25 | 2/6 | 0.72 | 1.00 |
| HFD | linear | Metabolome | Age | SVR | 0.2, 0.0635, 0.125 | 1/6 | 0.58 | 0.50 |
| HFD | linear | Metabolome | Age | RF | 0.2, 0.0025, 0.125 | 1/6 | 0.65 | 0.17 |
| HFD | linear | Metabolome | Age | XGBoost | 0.2, 0.0635, 2 | 1/6 | 0.70 | 0.00 |
| HFD | linear | Metabolome | Weight | ElasticNet | 0.2, 0.0635, 0.125 | 1/6 | 0.84 | 0.83 |
| HFD | linear | Metabolome | Weight | SVR | 0.2, 0.0635, 0.5 | 1/6 | 0.72 | 0.33 |

| Study | Weighting scheme | Omics | Response | Head | Modal best setting | Modal agreement | Mean Spearman | Top-decile rate |
|---|---|---|---|---|---|---|---|---|
| HFD | linear | Metabolome | Weight | RF | 0.2, 0.0025, 2 | 1/6 | 0.72 | 0.33 |
| HFD | linear | Metabolome | Weight | XGBoost | 0.4, 0.0635, 0.5 | 3/6 | 0.73 | 0.67 |
| HFD | linear | Microbiome | Age | ElasticNet | 0.2, 0.0635, 2 | 2/6 | 0.53 | 0.67 |
| HFD | linear | Microbiome | Age | SVR | 0.2, 0.0635, 2 | 1/6 | 0.48 | 0.33 |
| HFD | linear | Microbiome | Age | RF | 0.2, 0.0635, 2 | 1/6 | 0.53 | 0.50 |
| HFD | linear | Microbiome | Age | XGBoost | 0.4, 0.0025, 0.25 | 2/6 | 0.53 | 0.17 |
| HFD | linear | Microbiome | Weight | ElasticNet | 0.4, 0.0635, 0.125 | 2/6 | 0.72 | 0.50 |
| HFD | linear | Microbiome | Weight | SVR | 0.2, 0.0025, 0.5 | 1/6 | 0.72 | 0.33 |
| HFD | linear | Microbiome | Weight | RF | 0.2, 0.0025, 0.5 | 1/6 | 0.74 | 0.67 |
| HFD | linear | Microbiome | Weight | XGBoost | 0.6, 0.0126, 0.25 | 2/6 | 0.72 | 0.50 |
| HFD | exponential | Metabolome | Age | ElasticNet | 0.4, 0.0025, 0.125 | 1/6 | 0.63 | 0.33 |
| HFD | exponential | Metabolome | Age | SVR | 0.2, 0.0635, 0.125 | 1/6 | 0.67 | 0.67 |
| HFD | exponential | Metabolome | Age | RF | 0.2, 0.0126, 0.125 | 1/6 | 0.40 | 0.50 |
| HFD | exponential | Metabolome | Age | XGBoost | 0.2, 0.0635, 0.25 | 2/6 | 0.46 | 0.67 |
| HFD | exponential | Metabolome | Weight | ElasticNet | 0.2, 0.0635, 2 | 1/6 | 0.67 | 0.67 |
| HFD | exponential | Metabolome | Weight | SVR | 0.2, 0.0126, 1 | 1/6 | 0.57 | 0.67 |
| HFD | exponential | Metabolome | Weight | RF | 0.2, 0.0635, 2 | 1/6 | 0.43 | 0.50 |
| HFD | exponential | Metabolome | Weight | XGBoost | 0.2, 0.0025, 1 | 1/6 | 0.52 | 0.50 |
| HFD | exponential | Microbiome | Age | ElasticNet | 0.2, 0.0635, 1 | 2/6 | 0.61 | 0.67 |
| HFD | exponential | Microbiome | Age | SVR | 0.4, 0.0635, 0.125 | 2/6 | 0.68 | 0.83 |
| HFD | exponential | Microbiome | Age | RF | 0.4, 0.0635, 0.125 | 2/6 | 0.42 | 0.50 |
| HFD | exponential | Microbiome | Age | XGBoost | 0.4, 0.0635, 0.125 | 2/6 | 0.39 | 0.83 |
| HFD | exponential | Microbiome | Weight | ElasticNet | 0.2, 0.0635, 0.25 | 1/6 | 0.52 | 0.50 |
| HFD | exponential | Microbiome | Weight | SVR | 0.2, 0.32, 2 | 1/6 | 0.49 | 0.33 |
| HFD | exponential | Microbiome | Weight | RF | 0.6, 0.0025, 0.5 | 2/6 | 0.52 | 0.83 |
| HFD | exponential | Microbiome | Weight | XGBoost | 0.4, 0.0025, 0.125 | 2/6 | 0.53 | 0.50 |
| HFD | negative-log | Metabolome | Age | ElasticNet | 0.2, 0.0635, 0.25 | 2/6 | 0.73 | 1.00 |
| HFD | negative-log | Metabolome | Age | SVR | 0.2, 0.0635, 0.25 | 2/6 | 0.68 | 0.83 |
| HFD | negative-log | Metabolome | Age | RF | 0.2, 0.0126, 1 | 1/6 | 0.52 | 0.33 |
| HFD | negative-log | Metabolome | Age | XGBoost | 0.2, 0.0025, 2 | 1/6 | 0.59 | 0.33 |
| HFD | negative-log | Metabolome | Weight | ElasticNet | 0.2, 0.0635, 0.125 | 1/6 | 0.77 | 0.50 |
| HFD | negative-log | Metabolome | Weight | SVR | 0.2, 0.0126, 0.25 | 1/6 | 0.67 | 0.50 |
| HFD | negative-log | Metabolome | Weight | RF | 0.4, 0.0025, 1 | 2/6 | 0.61 | 0.17 |
| HFD | negative-log | Metabolome | Weight | XGBoost | 0.2, 0.0126, 0.25 | 1/6 | 0.61 | 0.00 |
| HFD | negative-log | Microbiome | Age | ElasticNet | 0.6, 0.32, 0.125 | 2/6 | 0.59 | 0.33 |
| HFD | negative-log | Microbiome | Age | SVR | 0.2, 0.0635, 0.125 | 1/6 | 0.53 | 0.50 |
| HFD | negative-log | Microbiome | Age | RF | 0.4, 0.0126, 2 | 2/6 | 0.44 | 0.33 |
| HFD | negative-log | Microbiome | Age | XGBoost | 0.2, 0.0635, 0.5 | 1/6 | 0.47 | 0.17 |
| HFD | negative-log | Microbiome | Weight | ElasticNet | 0.2, 0.0635, 0.5 | 1/6 | 0.46 | 0.50 |
| HFD | negative-log | Microbiome | Weight | SVR | 0.2, 0.0635, 0.5 | 1/6 | 0.48 | 0.50 |
| HFD | negative-log | Microbiome | Weight | RF | 0.2, 0.0025, 2 | 2/6 | 0.63 | 0.50 |
| HFD | negative-log | Microbiome | Weight | XGBoost | 0.2, 0.0025, 2 | 1/6 | 0.60 | 0.67 |

**Supplementary Table 4.** Prediction error under the additional simulation regimes. Best RMSE over the four regression heads on testing data for the additional simulation regimes, for the original data and for the MB-SupCon-cont embeddings using the three weighting methods. Values are means over 12 replicates.

| Regime | Omics | Original | Linear | Exponential | Negative-log |
|---|---|---|---|---|---|
| Baseline | Omics-1 | 10.097 | 9.080 | 9.309 | 9.123 |
| Baseline | Omics-2 | 9.438 | 8.848 | 9.308 | 9.080 |
| Noise | Omics-1 | 9.084 | 9.241 | 9.416 | 9.085 |
| Noise | Omics-2 | 8.967 | 8.907 | 9.424 | 9.186 |
| Single informative omics (Omics-1) | Omics-1 | 9.126 | 4.382 | 5.568 | 5.152 |
| Single informative omics (Omics-1) | Omics-2 | 11.932 | 12.955 | 12.848 | 12.960 |
| Single informative omics (Omics-2) | Omics-1 | 12.420 | 12.575 | 12.842 | 12.862 |
| Single informative omics (Omics-2) | Omics-2 | 7.414 | 3.545 | 5.761 | 4.993 |
| Sparse | Omics-1 | 9.732 | 8.411 | 8.660 | 8.566 |
| Sparse | Omics-2 | 9.101 | 8.681 | 9.023 | 8.685 |

**Supplementary Table 5.** Reverse PCA reconstruction quality on the real data. Reverse PCA reconstruction quality on the real data. For each omics data type and number of components k, the table reports the fraction of feature variance recovered by the low-rank reconstruction and the fraction of features whose reconstruction $R^2$ is below 0.5.

| Study | Omics | k | Variance recovered | Fraction of features with $R^2 < 0.5$ |
|---|---|---|---|---|
| T2D | Microbiome | 10 | 0.578 | 0.427 |
| T2D | Microbiome | 20 | 0.785 | 0.188 |
| T2D | Microbiome | 40 | 0.954 | 0.000 |
| T2D | Metabolome | 10 | 0.368 | 0.699 |
| T2D | Metabolome | 20 | 0.492 | 0.488 |
| T2D | Metabolome | 40 | 0.624 | 0.254 |
| HFD | Microbiome | 10 | 0.234 | 0.911 |
| HFD | Microbiome | 20 | 0.344 | 0.772 |
| HFD | Microbiome | 40 | 0.487 | 0.505 |
| HFD | Metabolome | 10 | 0.340 | 0.775 |
| HFD | Metabolome | 20 | 0.455 | 0.571 |
| HFD | Metabolome | 40 | 0.579 | 0.339 |

**Supplementary Table 6.** Full pairwise significance tests for every prediction comparison. Full pairwise significance tests for every prediction comparison in Table 1 and Supplementary Table 1. Each row compares MB-SupCon-cont, at the best weighting scheme and head for that cell, with one comparator at its own best head. The difference is the comparator RMSE minus the MB-SupCon-cont RMSE, so a positive value favours MB-SupCon-cont. The confidence interval is a 95% paired bootstrap interval for the mean difference, p is the paired Wilcoxon signed-rank p-value over the 12 splits, and q is the Benjamini-Hochberg adjusted p-value.

| Study | Omics | Response | MB-SupCon-cont (RMSE) | Comparator (RMSE) | Difference | 95% CI | p | q |
|---|---|---|---|---|---|---|---|---|
| T2D | Microbiome | Age | linear, RF (7.7085) | Baseline, XGBoost (7.3189) | -0.3896 | [-0.694, -0.086] | 0.0425 | 0.0588 |
| T2D | Microbiome | Age | linear, RF (7.7085) | CCA: RF (9.3040) | +1.5955 | [1.320, 1.861] | 0.0005 | 0.0011 |
| T2D | Microbiome | Age | linear, RF (7.7085) | Autoencoder: RF (9.0645) | +1.3560 | [1.006, 1.686] | 0.0005 | 0.0011 |
| T2D | Microbiome | BMI | exponential, SVR (2.9902) | Baseline, RF (2.8278) | -0.1624 | [-0.271, -0.044] | 0.0161 | 0.0232 |
| T2D | Microbiome | BMI | exponential, SVR (2.9902) | CCA: RF (3.7786) | +0.7884 | [0.645, 0.965] | 0.0005 | 0.0011 |
| T2D | Microbiome | BMI | exponential, SVR (2.9902) | Autoencoder: RF (3.5725) | +0.5823 | [0.443, 0.751] | 0.0005 | 0.0011 |
| T2D | Microbiome | SSPG | linear, RF (43.7351) | Baseline, XGBoost (40.9987) | -2.7364 | [-5.412, -0.272] | 0.1099 | 0.1413 |
| T2D | Microbiome | SSPG | linear, RF (43.7351) | CCA: RF (58.2669) | +14.5318 | [11.332, 17.749] | 0.0005 | 0.0011 |
| T2D | Microbiome | SSPG | linear, RF (43.7351) | Autoencoder: RF (52.0979) | +8.3628 | [5.147, 11.590] | 0.0024 | 0.0042 |
| T2D | Metabolome | Age | linear, RF (4.2379) | Baseline, XGBoost (4.9137) | +0.6758 | [0.423, 0.975] | 0.0005 | 0.0011 |
| T2D | Metabolome | Age | linear, RF (4.2379) | CCA: SVR (9.8050) | +5.5671 | [5.023, 6.171] | 0.0005 | 0.0011 |
| T2D | Metabolome | Age | linear, RF (4.2379) | Autoencoder: RF (8.1722) | +3.9343 | [3.550, 4.353] | 0.0005 | 0.0011 |
| T2D | Metabolome | BMI | negative-log, SVR (1.5221) | Baseline, XGBoost (1.7291) | +0.2070 | [0.091, 0.316] | 0.0093 | 0.0139 |
| T2D | Metabolome | BMI | negative-log, SVR (1.5221) | CCA: ElasticNet (4.0323) | +2.5102 | [2.356, 2.646] | 0.0005 | 0.0011 |
| T2D | Metabolome | BMI | negative-log, SVR (1.5221) | Autoencoder: SVR (2.9412) | +1.4191 | [1.255, 1.591] | 0.0005 | 0.0011 |
| T2D | Metabolome | SSPG | negative-log, XGBoost (20.0849) | Baseline, XGBoost (26.2918) | +6.2069 | [4.044, 8.279] | 0.0010 | 0.0019 |
| T2D | Metabolome | SSPG | negative-log, XGBoost (20.0849) | CCA: SVR (60.7627) | +40.6777 | [37.823, 43.568] | 0.0005 | 0.0011 |

| Study | Omics | Response | MB-SupCon-cont (RMSE) | Comparator (RMSE) | Difference | 95% CI | p | q |
|---|---|---|---|---|---|---|---|---|
| T2D | Metabolome | SSPG | negative-log, XGBoost (20.0849) | Autoencoder: RF (49.0548) | +28.9699 | [26.184, 31.739] | 0.0005 | 0.0011 |
| HFD | Microbiome | Age | linear, SVR (8.0994) | Baseline, XGBoost (7.1453) | -0.9541 | [-1.399, -0.522] | 0.0049 | 0.0078 |
| HFD | Microbiome | Age | linear, SVR (8.0994) | CCA: SVR (13.8022) | +5.7028 | [4.964, 6.357] | 0.0005 | 0.0010 |
| HFD | Microbiome | Age | linear, SVR (8.0994) | Autoencoder: RF (11.7820) | +3.6826 | [2.931, 4.519] | 0.0005 | 0.0010 |
| HFD | Microbiome | Weight | linear, SVR (8.0214) | Baseline, XGBoost (7.1812) | -0.8402 | [-1.164, -0.517] | 0.0010 | 0.0018 |
| HFD | Microbiome | Weight | linear, SVR (8.0214) | CCA: SVR (11.9269) | +3.9055 | [3.196, 4.554] | 0.0005 | 0.0010 |
| HFD | Microbiome | Weight | linear, SVR (8.0214) | Autoencoder: SVR (9.9513) | +1.9300 | [1.498, 2.401] | 0.0005 | 0.0010 |
| HFD | Metabolome | Age | negative-log, SVR (7.0759) | Baseline, XGBoost (8.3330) | +1.2571 | [0.936, 1.587] | 0.0005 | 0.0010 |
| HFD | Metabolome | Age | negative-log, SVR (7.0759) | CCA: RF (12.1549) | +5.0790 | [4.384, 5.699] | 0.0005 | 0.0010 |
| HFD | Metabolome | Age | negative-log, SVR (7.0759) | Autoencoder: RF (11.1029) | +4.0270 | [3.625, 4.478] | 0.0005 | 0.0010 |
| HFD | Metabolome | Weight | linear, XGBoost (6.3413) | Baseline, XGBoost (6.5848) | +0.2435 | [-0.119, 0.611] | 0.3804 | 0.3969 |
| HFD | Metabolome | Weight | linear, XGBoost (6.3413) | CCA: RF (8.8735) | +2.5322 | [2.120, 2.943] | 0.0005 | 0.0010 |
| HFD | Metabolome | Weight | linear, XGBoost (6.3413) | Autoencoder: RF (8.6241) | +2.2828 | [2.060, 2.534] | 0.0005 | 0.0010 |

**Supplementary Table 7.** Variance explained by the first two principal components. Share of variance explained by the first two principal components (PC1 and PC2 combined), for the original omics data and for the MB-SupCon-cont embeddings under the three weighting schemes, by study, omics data type, and response.

| Study | Omics | Response | Original | Linear | Exponential | Negative-log |
|---|---|---|---|---|---|---|
| T2D | Microbiome | Age | 0.223 | 0.878 | 0.617 | 0.553 |
| T2D | Microbiome | BMI | 0.227 | 0.935 | 0.689 | 0.630 |
| T2D | Microbiome | SSPG | 0.232 | 0.999 | 0.356 | 0.680 |
| T2D | Metabolome | Age | 0.143 | 0.829 | 0.452 | 0.542 |
| T2D | Metabolome | BMI | 0.151 | 0.840 | 0.657 | 0.592 |
| T2D | Metabolome | SSPG | 0.157 | 1.000 | 0.339 | 0.643 |
| HFD | Microbiome | Age | 0.088 | 1.000 | 0.779 | 0.524 |
| HFD | Microbiome | Weight | 0.088 | 0.974 | 1.000 | 0.966 |
| HFD | Metabolome | Age | 0.158 | 1.000 | 0.761 | 0.517 |
| HFD | Metabolome | Weight | 0.158 | 0.981 | 1.000 | 0.999 |

**Supplementary Table 8.** Weighting scheme performance against response range. Test RMSE of the three weighting schemes, ordered by response range, for the real cells. The random forest head is used.

| Study | Omics | Response | Response range | Linear | Exponential | Negative-log |
|---|---|---|---|---|---|---|
| T2D | Microbiome | BMI | 21.7 | 3.039 | 3.416 | 3.285 |
| T2D | Metabolome | BMI | 21.7 | 1.536 | 1.815 | 1.716 |
| T2D | Microbiome | Age | 49.3 | 7.709 | 8.211 | 7.727 |
| T2D | Metabolome | Age | 49.3 | 4.238 | 5.525 | 4.464 |
| HFD | Microbiome | Age | 51.0 | 8.449 | 8.917 | 8.717 |
| HFD | Metabolome | Age | 51.0 | 7.250 | 8.615 | 8.149 |
| HFD | Microbiome | Weight | 53.8 | 8.110 | 8.479 | 8.372 |
| HFD | Metabolome | Weight | 53.8 | 6.355 | 7.170 | 6.473 |
| T2D | Microbiome | SSPG | 236.0 | 43.735 | 45.378 | 44.662 |
| T2D | Metabolome | SSPG | 236.0 | 21.072 | 29.715 | 20.973 |

**Supplementary Table 9.** The validation-based selection rule per cell. Validation-based selection rule on the real data. For each study, omics data type, and response, the table gives the scheme the rule selects most often over the 12 splits, the mean test RMSE of the per-split selection (rule), the mean test RMSE of the best per-split choice (oracle), and the difference between them (regret).

| **Study** | **Omics** | **Response** | **Most-selected scheme** | **Rule (test RMSE)** | **Oracle (test RMSE)** | **Regret** |
|---|---|---|---|---|---|---|
| T2D | Microbiome | Age | linear | 7.825 | 7.507 | 0.319 |
| T2D | Microbiome | BMI | exponential | 3.045 | 2.896 | 0.149 |
| T2D | Microbiome | SSPG | linear | 44.895 | 42.017 | 2.878 |
| T2D | Metabolome | Age | linear | 4.439 | 4.145 | 0.294 |
| T2D | Metabolome | BMI | negative-log | 1.533 | 1.447 | 0.086 |
| T2D | Metabolome | SSPG | negative-log | 19.713 | 19.526 | 0.187 |
| HFD | Microbiome | Age | linear | 8.001 | 7.668 | 0.333 |
| HFD | Microbiome | Weight | linear | 7.927 | 7.754 | 0.174 |
| HFD | Metabolome | Age | negative-log | 7.117 | 6.855 | 0.262 |
| HFD | Metabolome | Weight | exponential | 6.543 | 6.316 | 0.227 |

**Supplementary Table 10.** Degradation of the three weighting schemes under response noise. Response-noise degradation of the three weighting schemes (random forest head). The columns give the test RMSE with no added noise and at the highest noise level (one standard deviation), and the absolute and relative increase.

| Study | Response | Omics | Scheme | RMSE (no noise) | RMSE (full noise) | Absolute increase | Relative increase |
|---|---|---|---|---|---|---|---|
| T2D | Age | Metabolome | linear | 4.238 | 7.208 | 2.971 | 0.701 |
| T2D | Age | Metabolome | exponential | 5.525 | 9.157 | 3.631 | 0.657 |
| T2D | Age | Metabolome | negative-log | 4.464 | 7.953 | 3.489 | 0.781 |
| T2D | BMI | Metabolome | linear | 1.536 | 3.006 | 1.470 | 0.957 |
| T2D | BMI | Metabolome | exponential | 1.815 | 3.920 | 2.105 | 1.160 |
| T2D | BMI | Metabolome | negative-log | 1.716 | 3.502 | 1.786 | 1.041 |
| T2D | SSPG | Metabolome | linear | 21.072 | 54.546 | 33.474 | 1.589 |
| T2D | SSPG | Metabolome | exponential | 29.715 | 46.145 | 16.430 | 0.553 |
| T2D | SSPG | Metabolome | negative-log | 20.973 | 52.108 | 31.135 | 1.485 |
| HFD | Age | Metabolome | linear | 7.250 | 10.831 | 3.581 | 0.494 |
| HFD | Age | Metabolome | exponential | 8.615 | 12.609 | 3.994 | 0.464 |
| HFD | Age | Metabolome | negative-log | 8.149 | 12.610 | 4.461 | 0.547 |
| HFD | Weight | Metabolome | linear | 6.355 | 10.272 | 3.917 | 0.616 |
| HFD | Weight | Metabolome | exponential | 7.170 | 10.195 | 3.025 | 0.422 |
| HFD | Weight | Metabolome | negative-log | 6.473 | 8.209 | 1.736 | 0.268 |

**Supplementary Table 11.** Mantel correlations and effective dimensionality of the embeddings for every study, omics data type, and response. Values are given for MB-SupCon-cont with the three weighting schemes, principal component analysis of the original data (raw PCA), and the autoencoder. For each cell the first row is the Mantel correlation, the mean correlation between the pairwise embedding distances and the pairwise response distances over the 12 splits, and the second row is the effective dimensionality, the participation ratio of the singular value spectrum of the embedding averaged over the 12 splits. A low participation ratio means the embedding variance is concentrated in a few directions and a high value means it is spread over many. All MB-SupCon-cont Mantel correlations are significant at $p < 0.001$.

| Study | Omics | Response | Measure | Linear | Exponential | Negative-log | Raw PCA | Autoencoder |
|---|---|---|---|---|---|---|---|---|
| T2D | Microbiome | Age | Mantel r | 0.267 | 0.215 | 0.621 | -0.001 | 0.016 |
| T2D | Microbiome | Age | Participation ratio | 1.73 | 3.75 | 4.18 | 7.59 | 5.13 |
| T2D | Microbiome | BMI | Mantel r | 0.336 | 0.353 | 0.607 | 0.001 | 0.016 |
| T2D | Microbiome | BMI | Participation ratio | 2.01 | 3.25 | 3.45 | 7.58 | 4.93 |
| T2D | Microbiome | SSPG | Mantel r | 0.733 | 0.202 | 0.565 | 0.067 | 0.074 |
| T2D | Microbiome | SSPG | Participation ratio | 1.02 | 7.34 | 2.79 | 7.52 | 5.23 |
| T2D | Metabolome | Age | Mantel r | 0.822 | 0.470 | 0.802 | 0.041 | 0.066 |
| T2D | Metabolome | Age | Participation ratio | 1.95 | 4.10 | 5.19 | 7.63 | 6.28 |
| T2D | Metabolome | BMI | Mantel r | 0.867 | 0.626 | 0.800 | 0.133 | 0.110 |
| T2D | Metabolome | BMI | Participation ratio | 1.58 | 3.38 | 4.17 | 7.63 | 6.22 |
| T2D | Metabolome | SSPG | Mantel r | 0.922 | 0.347 | 0.753 | 0.054 | 0.051 |
| T2D | Metabolome | SSPG | Participation ratio | 1.03 | 7.33 | 3.53 | 7.63 | 5.90 |
| HFD | Microbiome | Age | Mantel r | 0.829 | 0.643 | 0.746 | 0.208 | 0.198 |
| HFD | Microbiome | Age | Participation ratio | 1.07 | 2.55 | 3.51 | 8.36 | 4.91 |
| HFD | Microbiome | Weight | Mantel r | 0.540 | 0.543 | 0.523 | 0.006 | 0.011 |
| HFD | Microbiome | Weight | Participation ratio | 1.10 | 1.57 | 1.20 | 8.36 | 4.91 |
| HFD | Metabolome | Age | Mantel r | 0.878 | 0.644 | 0.791 | 0.117 | 0.198 |
| HFD | Metabolome | Age | Participation ratio | 1.06 | 2.54 | 3.35 | 6.81 | 2.08 |
| HFD | Metabolome | Weight | Mantel r | 0.750 | 0.752 | 0.716 | 0.086 | 0.050 |
| HFD | Metabolome | Weight | Participation ratio | 1.27 | 1.53 | 1.50 | 6.81 | 2.08 |

**Supplementary Table 12.** Leading features along each response axis. Leading features along the response axis, for every study, omics data type, and response: the full version of main-text Table 2, which lists only the five leading microbes per response. Features are ranked by their Spearman correlation with the MB-SupCon-cont response axis at the best weighting method for that cell, and the microbe and the metabolite of the same rank are shown side by side. In parentheses, each entry gives the correlation with the axis, the direct Spearman correlation with the response, and the number of the 12 splits in which the feature entered the top 20. The axis is fitted on the training samples and applied to the testing samples, and both correlations are computed there and averaged over the 12 splits. The naming rules are: a feature that was never identified keeps its place and is given by its molecular formula (T2D) or by its mass-to-charge ratio and retention time (HFD), and where several features resolve to the same taxon or compound only the highest ranked one is listed, so a cell can carry fewer than 20 rows.

| Study | Response | Rank | Microbe (axis, direct, splits) | Metabolite (axis, direct, splits) |
|---|---|---|---|---|
| T2D | Age | 1 | genus Blautia (+0.27, +0.24, 9/12) | dehydroepiandrosterone sulfate, DHEA-S (-0.53, -0.51, 12/12) |
| T2D | Age | 2 | genus Parabacteroides (+0.25, +0.15, 8/12) | C6H10O4S (+0.49, +0.43, 12/12) |
| T2D | Age | 3 | family Lachnospiraceae (+0.22, +0.16, 7/12) | N6-carbamoyl-L-threonyladenosine (+0.44, +0.33, 10/12) |
| T2D | Age | 4 | genus Clostridium IV (+0.22, +0.21, 8/12) | C30H46O10 (+0.43, +0.37, 9/12) |
| T2D | Age | 5 | genus Prevotella (-0.20, -0.10, 7/12) | C7H14O7 (+0.42, +0.34, 9/12) |
| T2D | Age | 6 | genus Pseudoflavonifractor (+0.20, +0.20, 7/12) | N-acetyl-1-methylhistidine (+0.42, +0.39, 6/12) |
| T2D | Age | 7 | genus Clostridium XlVb (+0.20, +0.12, 7/12) | L-cystine (+0.40, +0.32, 6/12) |
| T2D | Age | 8 | unclassified Lachnospiraceae (+0.19, +0.12, 7/12) | 5-methyluridine (ribothymidine) (+0.40, +0.32, 7/12) |
| T2D | Age | 9 | family Acidaminococcaceae (+0.18, +0.17, 6/12) | C27H42O11 (+0.40, +0.33, 8/12) |
| T2D | Age | 10 | family Porphyromonadaceae (+0.18, +0.08, 7/12) | pregnanolone sulfate (-0.40, -0.38, 8/12) |
| T2D | Age | 11 | genus Lachnospiracea incertae sedis (+0.17, +0.15, 5/12) | pantothenic acid (+0.40, +0.37, 7/12) |
| T2D | Age | 12 | class Gammaproteobacteria (-0.17, -0.19, 6/12) | dihydroxyvitamin D3 (-0.40, -0.38, 7/12) |
| T2D | Age | 13 | genus Clostridium XlVa (+0.16, +0.11, 5/12) | N2,N2-Dimethylguanosine (+0.39, +0.31, 6/12) |
| T2D | Age | 14 | family Prevotellaceae (-0.15, -0.08, 5/12) | gamma-glutamylphenylalanine (+0.39, +0.32, 7/12) |
| T2D | Age | 15 | family Veillonellaceae (-0.15, -0.10, 5/12) | pyridoxic acid (+0.39, +0.36, 7/12) |
| T2D | Age | 16 | genus Anaerotruncus (+0.14, +0.12, 4/12) | C21H34O6S (-0.39, -0.38, 7/12) |
| T2D | Age | 17 | class Clostridia (+0.13, +0.07, 4/12) | C5:0,DC acylcarnitine (+0.39, +0.35, 5/12) |

| Study | Response | Rank | Microbe<br>(axis, direct, splits) | Metabolite<br>(axis, direct, splits) |
|---|---|---|---|---|
| T2D | Age | 18 | class Bacteroidia (-0.13, -0.07, 3/12) | orotidine (+0.38, +0.31, 5/12) |
| T2D | Age | 19 | order Bacteroidales (-0.13, -0.07, 3/12) | C7H15N3O2 (+0.38, +0.31, 5/12) |
| T2D | Age | 20 | order Clostridiales (+0.13, +0.07, 5/12) | C25H36O12 (+0.37, +0.34, 4/12) |
| T2D | BMI | 1 | genus Odoribacter (-0.29, -0.22, 11/12) | C20:0,2OH fatty acid (-0.49, -0.46, 12/12) |
| T2D | BMI | 2 | genus Barnesiella (-0.28, -0.27, 11/12) | C6H12O5 (+0.47, +0.44, 11/12) |
| T2D | BMI | 3 | genus Clostridium XlVa (+0.26, +0.21, 9/12) | 5-alpha-androstan-3-alpha,17-alpha-diol monosulfate (-0.46, -0.45, 11/12) |
| T2D | BMI | 4 | family Streptococcaceae (+0.21, +0.15, 6/12) | dihydroxyvitamin D3 (-0.44, -0.41, 11/12) |
| T2D | BMI | 5 | unclassified Bacteria (-0.20, -0.13, 6/12) | 2-hydroxyisocaproic acid (-0.42, -0.39, 9/12) |
| T2D | BMI | 6 | genus Clostridium XlVb (+0.20, +0.12, 5/12) | phenyllactate (-0.42, -0.39, 9/12) |
| T2D | BMI | 7 | genus Streptococcus (+0.19, +0.14, 5/12) | C7H10O6 (+0.41, +0.38, 8/12) |
| T2D | BMI | 8 | genus Clostridium XI (-0.19, -0.22, 7/12) | indolelactic acid (-0.39, -0.35, 8/12) |
| T2D | BMI | 9 | family Peptostreptococcaceae (-0.19, -0.22, 7/12) | pregnanolone sulfate (-0.39, -0.36, 7/12) |
| T2D | BMI | 10 | genus Butyricimonas (-0.19, -0.18, 7/12) | 3-Methyl-2-oxovaleric acid (-0.39, -0.36, 7/12) |
| T2D | BMI | 11 | order Lactobacillales (+0.18, +0.12, 5/12) | dehydroepiandrosterone sulfate, DHEA-S (-0.37, -0.35, 8/12) |
| T2D | BMI | 12 | class Bacilli (+0.18, +0.12, 6/12) | androsterone sulfate (-0.37, -0.36, 7/12) |
| T2D | BMI | 13 | unclassified Clostridiales (-0.17, -0.15, 4/12) | pseudouridine (+0.37, +0.35, 5/12) |
| T2D | BMI | 14 | genus Erysipelotrichaceae incertae sedis (+0.16, +0.11, 5/12) | C16:1 fatty acid (+0.37, +0.34, 4/12) |
| T2D | BMI | 15 | family Rikenellaceae (-0.15, -0.12, 3/12) | L-_-Hydroxyisovaleric acid (-0.37, -0.36, 4/12) |
| T2D | BMI | 16 | | C21H34O6S (-0.35, -0.31, 4/12) |
| T2D | BMI | 17 | | C14H22N2O9 (-0.34, -0.34, 3/12) |
| T2D | BMI | 18 | | C30H30O5S (-0.33, -0.30, 3/12) |
| T2D | BMI | 19 | | C11H11NO3 (-0.33, -0.30, 3/12) |
| T2D | SSPG | 1 | unclassified Clostridiales (-0.49, -0.46, 12/12) | C20:0,2OH fatty acid (-0.48, -0.44, 12/12) |
| T2D | SSPG | 2 | unclassified Bacteria (-0.48, -0.37, 12/12) | 4-formyl indole (-0.42, -0.39, 9/12) |
| T2D | SSPG | 3 | unclassified Lachnospiraceae (-0.38, -0.29, 10/12) | phenyllactate (-0.41, -0.36, 8/12) |
| T2D | SSPG | 4 | family Clostridiaceae 1 (-0.37, -0.37, 10/12) | androsterone glucuronide (-0.41, -0.37, 9/12) |

| Study | Response | Rank | Microbe (axis, direct, splits) | Metabolite (axis, direct, splits) |
|---|---|---|---|---|
| T2D | SSPG | 5 | genus Clostridium sensu stricto (-0.37, -0.37, 11/12) | C14H14O3 (+0.38, +0.35, 8/12) |
| T2D | SSPG | 6 | unclassified Firmicutes (-0.37, -0.32, 9/12) | methyl-4-hydroxybenzoate sulfate (-0.38, -0.38, 8/12) |
| T2D | SSPG | 7 | genus Pseudoflavonifractor (-0.35, -0.30, 6/12) | C8:1 acylcarnitine (+0.38, +0.32, 6/12) |
| T2D | SSPG | 8 | genus Odoribacter (-0.33, -0.27, 8/12) | C20:4,DC fatty acid (-0.38, -0.33, 5/12) |
| T2D | SSPG | 9 | unclassified Ruminococcaceae (-0.32, -0.33, 9/12) | hippuric acid (-0.38, -0.37, 5/12) |
| T2D | SSPG | 10 | genus Coprococcus (-0.32, -0.30, 7/12) | C7H10N2O3 (-0.37, -0.31, 5/12) |
| T2D | SSPG | 11 | genus Barnesiella (-0.30, -0.30, 6/12) | C24H38O4 (+0.37, +0.34, 6/12) |
| T2D | SSPG | 12 | genus Anaerovorax (-0.30, -0.21, 7/12) | C14:0,DC fatty acid (-0.34, -0.30, 4/12) |
| T2D | SSPG | 13 | | C10:0,OH fatty acid (-0.34, -0.31, 4/12) |
| T2D | SSPG | 14 | | eugenol sulfate (-0.34, -0.32, 6/12) |
| T2D | SSPG | 15 | | C5:1 acylcarnitine (-0.33, -0.26, 3/12) |
| T2D | SSPG | 16 | | C10:3 fatty acid (+0.33, +0.26, 6/12) |
| T2D | SSPG | 17 | | arabonate |
| T2D | SSPG | 18 | | indolelactic acid (-0.32, -0.28, 3/12) |
| T2D | SSPG | 19 | | phenylalanylphenylalanine (-0.32, -0.27, 1/12) |
| T2D | SSPG | 20 | | C10:3 acylcarnitine (+0.32, +0.28, 3/12) |
| HFD | Age | 1 | family Muribaculaceae, S24-7 (-0.45, -0.43, 8/12) | m/z 334.294, RT 4.79 (+0.61, +0.42, 7/12) |
| HFD | Age | 2 | genus Anaeroplasma (-0.42, -0.38, 8/12) | PubChem CID 45360040 (C22H30O6) (+0.59, +0.49, 5/12) |
| HFD | Age | 3 | genus Bacteroides (-0.39, -0.36, 5/12) | m/z 426.321, RT 5.69 (+0.57, +0.43, 2/12) |
| HFD | Age | 4 | order Rickettsiales (-0.38, -0.38, 6/12) | m/z 442.315, RT 4.98 (+0.56, +0.46, 3/12) |
| HFD | Age | 5 | unclassified Bacteria (-0.38, -0.37, 5/12) | m/z 549.401, RT 5.13 (+0.55, +0.43, 1/12) |
| HFD | Age | 6 | family Lachnospiraceae (-0.38, -0.35, 6/12) | m/z 311.197, RT 3.67 (+0.55, +0.39, 2/12) |
| HFD | Age | 7 | family Ruminococcaceae (-0.37, -0.28, 5/12) | m/z 313.213, RT 4.23 (+0.55, +0.39, 2/12) |
| HFD | Age | 8 | class Betaproteobacteria (-0.34, -0.34, 4/12) | m/z 544.339, RT 7.33 (+0.54, +0.44, 2/12) |
| HFD | Age | 9 | | m/z 425.240, RT 3.33 (+0.54, +0.39, 4/12) |

| Study | Response | Rank | Microbe (axis, direct, splits) | Metabolite (axis, direct, splits) |
|---|---|---|---|---|
| HFD | Age | 10 | | m/z 570.349, RT 3.48 (+0.54, +0.38, 2/12) |
| HFD | Age | 11 | | m/z 476.265, RT 6.49 (+0.54, +0.46, 3/12) |
| HFD | Age | 12 | | m/z 424.304, RT 4.31 (+0.54, +0.38, 4/12) |
| HFD | Age | 13 | | m/z 424.306, RT 5.23 (+0.54, +0.42, 3/12) |
| HFD | Age | 14 | | m/z 434.244, RT 6.49 (+0.54, +0.47, 2/12) |
| HFD | Weight | 1 | family Lachnospiraceae (+0.53, +0.45, 9/12) | m/z 374.277, RT 6.53 (+0.69, +0.58, 5/12) |
| HFD | Weight | 2 | genus Candidatus Arthromitus (-0.49, -0.42, 8/12) | m/z 435.310, RT 8.15 (+0.69, +0.54, 6/12) |
| HFD | Weight | 3 | family Muribaculaceae, S24-7 (-0.49, -0.47, 6/12) | m/z 365.242, RT 5.05 (+0.69, +0.59, 3/12) |
| HFD | Weight | 4 | species Ruminococcus gnavus (+0.49, +0.35, 6/12) | glycodeoxycholic acid (+0.68, +0.59, 4/12) |
| HFD | Weight | 5 | family Erysipelotrichaceae (+0.48, +0.45, 7/12) | m/z 345.183, RT 4.01 (+0.68, +0.55, 4/12) |
| HFD | Weight | 6 | order Clostridiales (-0.45, -0.42, 5/12) | m/z 435.311, RT 8.85 (+0.68, +0.54, 4/12) |
| HFD | Weight | 7 | genus Oscillospira (-0.44, -0.33, 5/12) | m/z 412.237, RT 4.18 (+0.68, +0.55, 3/12) |
| HFD | Weight | 8 | genus Ruminococcus (+0.43, +0.32, 3/12) | m/z 440.295, RT 3.50 (+0.67, +0.57, 3/12) |
| HFD | Weight | 9 | class Betaproteobacteria (-0.43, -0.40, 5/12) | m/z 530.298, RT 3.70 (+0.67, +0.57, 3/12) |
| HFD | Weight | 10 | genus Prevotella (-0.43, -0.37, 4/12) | chenodeoxycholic acid (+0.66, +0.57, 3/12) |
| HFD | Weight | 11 | | m/z 521.395, RT 5.06 (+0.66, +0.57, 3/12) |
| HFD | Weight | 12 | | m/z 314.217, RT 4.22 (+0.66, +0.55, 3/12) |
| HFD | Weight | 13 | | m/z 367.165, RT 4.01 (+0.66, +0.54, 3/12) |
| HFD | Weight | 14 | | m/z 377.200, RT 4.19 (+0.66, +0.55, 1/12) |
| HFD | Weight | 15 | | m/z 348.744, RT 8.81 (+0.66, +0.62, 4/12) |
| HFD | Weight | 16 | | m/z 355.263, RT 6.52 (+0.66, +0.56, 3/12) |

**Supplementary Table 13.** Embedding-based interpretation against its three controls. Embedding-based biological interpretation and its controls. For each study, omics data type, and response, at its best weighting scheme, each entry gives the correlation R between the held-out response and the embedding response axis, followed by the number of the direct top 20 features that the axis recovers. The response axis is fitted on the training samples and applied to the testing samples, and both rankings are computed on the testing samples and averaged over the 12 splits. The shuffled-response encoder is MB-SupCon-cont retrained with permuted responses. The last column is the overlap expected by chance (hypergeometric mean), which reflects the number of features in each omics data type. The rank correlation over all features is given in the source file.

| Study | Omics | Response | Best scheme | MB-SupCon-cont | Autoencoder | Raw PCA | Shuffled response | Chance |
|---|---|---|---|---|---|---|---|---|
| T2D | Microbiome | Age | linear | 0.52, 9/20 | 0.14, 3/20 | 0.10, 4/20 | 0.14, 4/20 | 4.17/20 |
| T2D | Microbiome | BMI | exponential | 0.58, 10/20 | 0.29, 5/20 | 0.26, 4/20 | 0.10, 7/20 | 4.17/20 |
| T2D | Microbiome | SSPG | linear | 0.71, 17/20 | 0.42, 13/20 | 0.44, 14/20 | 0.20, 12/20 | 4.17/20 |
| T2D | Metabolome | Age | linear | 0.87, 14/20 | 0.45, 9/20 | 0.44, 11/20 | 0.13, 4/20 | 0.55/20 |
| T2D | Metabolome | BMI | negative-log | 0.91, 17/20 | 0.52, 9/20 | 0.61, 13/20 | 0.17, 5/20 | 0.55/20 |
| T2D | Metabolome | SSPG | negative-log | 0.93, 15/20 | 0.47, 7/20 | 0.57, 5/20 | 0.16, 1/20 | 0.55/20 |
| HFD | Microbiome | Age | linear | 0.79, 15/20 | 0.44, 7/20 | 0.62, 5/20 | 0.09, 1/20 | 0.44/20 |
| HFD | Microbiome | Weight | linear | 0.68, 14/20 | 0.47, 9/20 | 0.54, 8/20 | 0.25, 6/20 | 0.44/20 |
| HFD | Metabolome | Age | negative-log | 0.85, 11/20 | 0.53, 0/20 | 0.60, 2/20 | 0.24, 0/20 | 0.03/20 |
| HFD | Metabolome | Weight | linear | 0.82, 9/20 | 0.59, 1/20 | 0.59, 4/20 | 0.34, 0/20 | 0.03/20 |

**Supplementary Tables 14 to 23.** Per-head prediction results. For each real study, the mean testing RMSEs for predictions based on the original omics data and on the MB-SupCon-cont embeddings are listed here. The results are summarized for each response, each omics data type (microbiome and metabolome), and each prediction head (ElasticNet, SVR, RF, and XGBoost). The dimension of the embedding is 10. The "Original" column is the prediction on the original omics data with that head. The Linear, Exponential, and Negative-log columns are the predictions on the embeddings learned with each weighting method. Values are means over 12 random training-validation-testing splits.

**Supplementary Table 14.** Average RMSEs on testing data for predicting Age based on microbiome data and microbiome embeddings from MB-SupCon-cont (T2D study)

| Predictor | Original | Linear | Exponential | Negative-log |
|---|---|---|---|---|
| ElasticNet | 9.4462 | 8.4268 | 8.6322 | 7.9730 |
| SVR | 9.6207 | 7.7948 | 8.2832 | 7.8399 |
| RF | 7.8069 | 7.7085 | 8.2113 | 7.7273 |
| XGBoost | 8.1214 | 7.7782 | 8.1742 | 7.8869 |

**Supplementary Table 15.** Average RMSEs on testing data for predicting Age based on metabolome data and metabolome embeddings from MB-SupCon-cont (T2D study)

| Predictor | Original | Linear | Exponential | Negative-log |
|---|---|---|---|---|
| ElasticNet | 6.3917 | 5.4045 | 6.8846 | 5.1392 |
| SVR | 8.8224 | 4.5337 | 5.5001 | 4.9757 |
| RF | 5.6916 | 4.2379 | 5.5255 | 4.4643 |
| XGBoost | 5.7289 | 4.3031 | 5.1786 | 4.4586 |

**Supplementary Table 16.** Average RMSEs on testing data for predicting BMI based on microbiome data and microbiome embeddings from MB-SupCon-cont (T2D study)

| Predictor | Original | Linear | Exponential | Negative-log |
|---|---|---|---|---|
| ElasticNet | 3.8306 | 3.2313 | 3.2461 | 3.0578 |
| SVR | 3.5244 | 3.0339 | 2.9902 | 2.9928 |
| RF | 2.9652 | 3.0389 | 3.4163 | 3.2851 |
| XGBoost | 3.1530 | 3.0739 | 3.2573 | 3.2417 |

**Supplementary Table 17.** Average RMSEs on testing data for predicting BMI based on metabolome data and metabolome embeddings from MB-SupCon-cont (T2D study)

| Predictor | Original | Linear | Exponential | Negative-log |
|---|---|---|---|---|
| ElasticNet | 2.8708 | 2.0467 | 2.4345 | 2.0463 |
| SVR | 2.8792 | 1.5781 | 1.6323 | 1.5221 |
| RF | 2.1848 | 1.5360 | 1.8150 | 1.7159 |
| XGBoost | 2.1154 | 1.5647 | 1.7196 | 1.5983 |

**Supplementary Table 18.** Average RMSEs on testing data for predicting SSPG based on microbiome data and microbiome embeddings from MB-SupCon-cont (T2D study)

| Predictor | Original | Linear | Exponential | Negative-log |
|---|---|---|---|---|
| ElasticNet | 48.1111 | 44.2481 | 48.5568 | 43.8193 |
| SVR | 59.0257 | 44.1560 | 53.4439 | 45.4932 |
| RF | 41.9548 | 43.7351 | 45.3779 | 44.6617 |
| XGBoost | 44.1595 | 44.4472 | 48.6307 | 45.5813 |

**Supplementary Table 19.** Average RMSEs on testing data for predicting SSPG based on metabolome data and metabolome embeddings from MB-SupCon-cont (T2D study)

| Predictor | Original | Linear | Exponential | Negative-log |
|---|---|---|---|---|
| ElasticNet | 41.7313 | 23.4551 | 38.6399 | 25.1788 |
| SVR | 59.3345 | 27.3099 | 48.2636 | 29.2506 |
| RF | 34.4950 | 21.0723 | 29.7155 | 20.9729 |
| XGBoost | 33.5242 | 21.5815 | 30.5596 | 20.0849 |

**Supplementary Table 20.** Average RMSEs on testing data for predicting Age based on microbiome data and microbiome embeddings from MB-SupCon-cont (HFD study)

| Predictor | Original | Linear | Exponential | Negative-log |
|---|---|---|---|---|
| ElasticNet | 8.6952 | 8.3708 | 8.8320 | 8.5939 |
| SVR | 12.5909 | 8.0994 | 8.1475 | 8.1510 |
| RF | 8.3439 | 8.4488 | 8.9169 | 8.7172 |
| XGBoost | 8.4767 | 8.5934 | 9.2279 | 8.8802 |

**Supplementary Table 21.** Average RMSEs on testing data for predicting Age based on metabolome data and metabolome embeddings from MB-SupCon-cont (HFD study)

| Predictor | Original | Linear | Exponential | Negative-log |
|---|---|---|---|---|
| ElasticNet | 8.9193 | 7.4206 | 8.3830 | 7.5464 |
| SVR | 12.8361 | 7.2369 | 7.5197 | 7.0759 |
| RF | 9.1652 | 7.2499 | 8.6151 | 8.1493 |
| XGBoost | 9.3198 | 7.3178 | 8.7177 | 7.5108 |

**Supplementary Table 22.** Average RMSEs on testing data for predicting Weight based on microbiome data and microbiome embeddings from MB-SupCon-cont (HFD study)

| Predictor | Original | Linear | Exponential | Negative-log |
|---|---|---|---|---|
| ElasticNet | 8.8105 | 8.6272 | 8.5331 | 8.7479 |
| SVR | 10.5297 | 8.0214 | 8.1225 | 8.3642 |
| RF | 7.5095 | 8.1102 | 8.4795 | 8.3720 |
| XGBoost | 7.8293 | 8.3068 | 8.6621 | 8.5798 |

**Supplementary Table 23.** Average RMSEs on testing data for predicting Weight based on metabolome data and metabolome embeddings from MB-SupCon-cont (HFD study)

| Predictor | Original | Linear | Exponential | Negative-log |
|---|---|---|---|---|
| ElasticNet | 9.3766 | 7.2923 | 7.0962 | 7.4036 |
| SVR | 9.7355 | 6.3998 | 6.6964 | 6.6872 |
| RF | 6.9497 | 6.3552 | 7.1696 | 6.4727 |
| XGBoost | 7.2810 | 6.3413 | 7.0201 | 6.5033 |

## References

Benjamini Y, Hochberg Y. 1995. Controlling the False Discovery Rate: A Practical and Powerful Approach to Multiple Testing. Journal of the Royal Statistical Society: Series B (Methodological).57:289–300.

Chen T, Guestrin C. 2016. XGBoost: A Scalable Tree Boosting System. Proceedings of the 22nd ACM SIGKDD International Conference on Knowledge Discovery and Data Mining.

HOTELLING H. 1936. RELATIONS BETWEEN TWO SETS OF VARIATES. Biometrika.28:321–377.

Mantel N. 1967. The Detection of Disease Clustering and a Generalized Regression Approach. Cancer Research.27:209–220.

Morton JT, Aksenov AA, Nothias LF, Foulds JR, Quinn RA, Badri MH, Swenson TL, Van Goethem MW, Northen TR, Vazquez-Baeza Y, et al. 2019. Learning representations of microbe-metabolite interactions. Nat Methods. Dec;16:1306–1314. Epub 20191104.

Pedregosa F, Varoquaux G, Gramfort A, Michel V, Thirion B, Grisel O, Blondel M, Prettenhofer P, Weiss R, Dubourg V. 2011. Scikit-learn: Machine learning in Python. the Journal of machine Learning research.12:2825–2830.

Zhou W, Sailani MR, Contrepois K, Zhou Y, Ahadi S, Leopold SR, Zhang MJ, Rao V, Avina M, Mishra T, et al. 2019. Longitudinal multi-omics of host-microbe dynamics in prediabetes. Nature. May;569:663–671. Epub 20190529.
